# Comprehensive transcriptomics and proteomics analyses of pollinated and parthenocarpic litchi (*Litchi chinensis* Sonn.) fruits during early development

**DOI:** 10.1038/s41598-017-05724-z

**Published:** 2017-07-14

**Authors:** Wei Liu, Maoshan Chen, Lijun Bai, Zhenhua Zhuang, Chao Fan, Nonghui Jiang, Junsheng Zhao, Shuaipeng Ma, Xu Xiang

**Affiliations:** 1 Institute of Fruit Tree Research, Guangdong Academy of Agricultural Sciences, Guangzhou, 510640 China; 2 Key Laboratory of South Subtropical Fruit Biology and Genetic Resource Utilization, Ministry of Agriculture, Guangzhou, 510640 China; 3 Guangdong Provincial Key Laboratory of Tropical and Subtropical Fruit Tree Research, Guangzhou, 510640 China; 4 Department of Biochemistry and Genetics, La Trobe Institute for Molecular Science (LIMS), La Trobe University, Melbourne, Victoria, 3086 Australia; 5 Chengdu Life Baseline Technology, Chengdu, 610041 China; 6 Institute of Fruit Science in Maoming, Maoming, 525000 China; 7 Guangzhou Experiment Station of Chinese Academy of Tropical Agricultural Sciences, Guangzhou, 510140 China

## Abstract

Litchi (*Litchi chinensis* Sonn.) is an important fruit that is widely cultivated in tropical and subtropical areas. In this study, we used RNA-Seq and iTRAQ technologies to compare the transcriptomes and proteomes of pollinated (polLFs) and parthenocarpic (parLFs) litchi fruits during early development (1 day, 2 days, 4 days and 6 days). We identified 4,864 DEGs in polLFs and 3,672 in parLFs, of which 2,835 were shared and 1,051 were specifically identified in parLFs. Compared to po1LFs, 768 DEGs were identified in parLFs. iTRAQ analysis identified 551 DEPs in polLFs and 1,021 in parLFs, of which 305 were shared and 526 were exclusively identified in parLFs. We found 1,127 DEPs in parLFs compared to polLFs at different stages. Further analysis revealed some DEGs/DEPs associated with abscisic acid, auxin, ethylene, gibberellin, heat shock protein (HSP), histone, ribosomal protein, transcription factor and zinc finger protein (ZFP). WGCNA identified a large set of co-expressed genes/proteins in polLFs and parLFs. In addition, a cross-comparison of transcriptomic and proteomic data identified 357 consistent DEGs/DEPs in polLFs and parLFs. This is the first time that protein/gene changes have been studied in polLFs and parLFs, and the findings improve our understanding of litchi parthenocarpy.

## Introduction

Litchi (*Litchi chinensis* Sonn.), a plant from the Sapindaceae family, is one of the most important tropical and subtropical fruit trees and wildly cultivated in South China and other areas in the world. It has been cultivated in China for over 2,300 years and it is becoming more and more popular all over the world due to its attractive red color, sweet taste and nutritional value. Considering its significant economic contribution, it is necessary to improve the fruit quality and study the gene expression changes in litchi fruit development. The phenomenon of natural parthenocarpy is one of the invaluable traits found in litchi, which is supposed to overcome the alternate bearing that often occurs from poor pollination in the litchi industry, and it is also an important pathway for producing seedless (chicken-tongue) fruit, which is an important commercial trait for fresh, quality litchi.

Fruit development, which is defined as the differentiation of a pre-existing organ, is a key process in the plant life cycle and can be divided into three phases^1^: (1) ovary development, fertilization, and fruit set; (2) cell division, seed formation, and early embryo development; (3) cell expansion and embryo maturation. In the first step, fruit set is dependent on successful pollination and fertilization, or else the carpel will senesce. However, fruit set can be affected by environmental conditions such as light and temperature^2^. It has been shown that some fruits can be produced without seeds, also called parthenocarpy, by natural mutations, environmental factors or hormone treatments^3^. In plants, parthenocarpy can be induced by the exogenous hormone auxin and gibberellin (GA)^4^. Three GAs (GA3, GA4, GA7) have been reported to induce the parthenocarpy in Rosaceae species including apple^5^, loquat^6^ and peach^7^. GA signalling can be regulated by two genes – GID1 (gibberellin insensitive DWARF 1), which directly binds to GA^8^ and RGA (DELLA protein) that is a transcriptional repressor of GA response^9^. Auxin treatment can affect the expression of numerous genes, including two major families – AUXIN RESPONSIVE FACTORs (ARFs) and AUXIN-INDUCED proteins (Aux/IAAs)^10, 11^. By interacting with the ARF dimerization domain, Aux/IAA proteins suppress the expression of genes in the auxin signalling pathway^12^. In addition, ARFs can interact with the auxin responsive elements (AuxREs) in the promoter region of auxin responsive genes through an amino-terminus DNA-binding domain^13^. However, significant levels of auxin can direct the Aux/IAA proteins to proteasomal degradation, which leads to the release of ARFs and regulates downstream gene expression^14^. In Arabidopsis and tomato, auxin-associated proteins, such as IAA9^15^, ARF2, ARF7^16^, ARF8^17^ and ARF9^18^, have been characterized as related to parthenocarpic fruit production. More recently, ARF9 was characterized to regulate cell division activity during early fruit development in tomato^19^.

Next-generation RNA sequencing (RNA-Seq) and ‘isobaric tags for relative and absolute quantification’ (iTRAQ) proteomics are well-developed technologies to globally profile the expression of genes and proteins, respectively, in specific bio samples^20–22^. In tomato, RNA-Seq was applied to compare the transcriptomes of pollinated and 2,4-D/GA_3_-treated ovaries and found important regulatory pathways during pollinated and parthenocarpic fruit set, such as the activation of carbohydrate metabolism, cell division and expansion, and the down-regulation of MADS-box^18^. Transcriptome analysis of the fruit setting induced by GA3 during the early fruit development in triploid loquat identified various differentially expressed genes (DEGs) including 5 auxin genes and four transcription factors (TFs)^23^. Although there are few iTRAQ studies on parthenocarpy, it has been widely used to study the protein expression and functions of plants, including grape fruit development and ripening^24^, *Flammulina velutipes* mycelia in response to cold stress^25^, oriental melon fruit quality at different developmental stages^26^ and maize gain development^27^. However, the protein and gene expression changes of pollinated and parthenocarpic litchi fruit during the early development are still unknown.

Among current litchi cultivars, some have been proven to have an ability for parthenocarpy, including ‘Hexiachuan’, ‘Maohongnuo’, ‘Jiaxiangli’ and ‘Jiagualu’^28^. Among them, the ovary size, weight of the fresh fruit and endogenous hormone levels have been well studied in ‘Hexiachuan’ with or without pollination^29–32^. In addition, these characteristics of parthenocarpic litchi, which can provide a rich harvest, even in the rainy season without fertilization, are of great importance because they can guide and benefit the litchi industry when the mechanism of parthenocarpy is understood. In this study, we used RNA-Seq and iTRAQ proteomics technologies to study the gene and protein expression changes in pollinated and parthenocarpic ‘Hexiachuan’ litchi fruits during early development, respectively. Differential expression analysis revealed several genes were involved in litchi fruit development, including abscisic acid (ABA), auxin, ethylene, GA, heat shock proteins (HSPs), histone, ribosomal proteins (RPs), TFs and zinc finger proteins (ZFPs). Furthermore, some of the genes were found dysregulated in a comparison of pollinated and parthenocarpic litchi fruits at different developmental stages, including auxin, GA, HSPs, histone, RPs, ZFPs and various TFs. This is the first time that global gene and protein expression changes in litchi fruit development, especially in parthenocarpic litchi fruits, has been studied. Our findings provide a valuable resource on parthenocarpy-related genes and proteins, which will benefit researchers in this field. Also, this study will improve our understanding of parthenocarpy, will help overcome the alternate bearing and will improve litchi fruit quality.

## Results

### Overview of litchi fruit transcriptome

**Figure 1:**
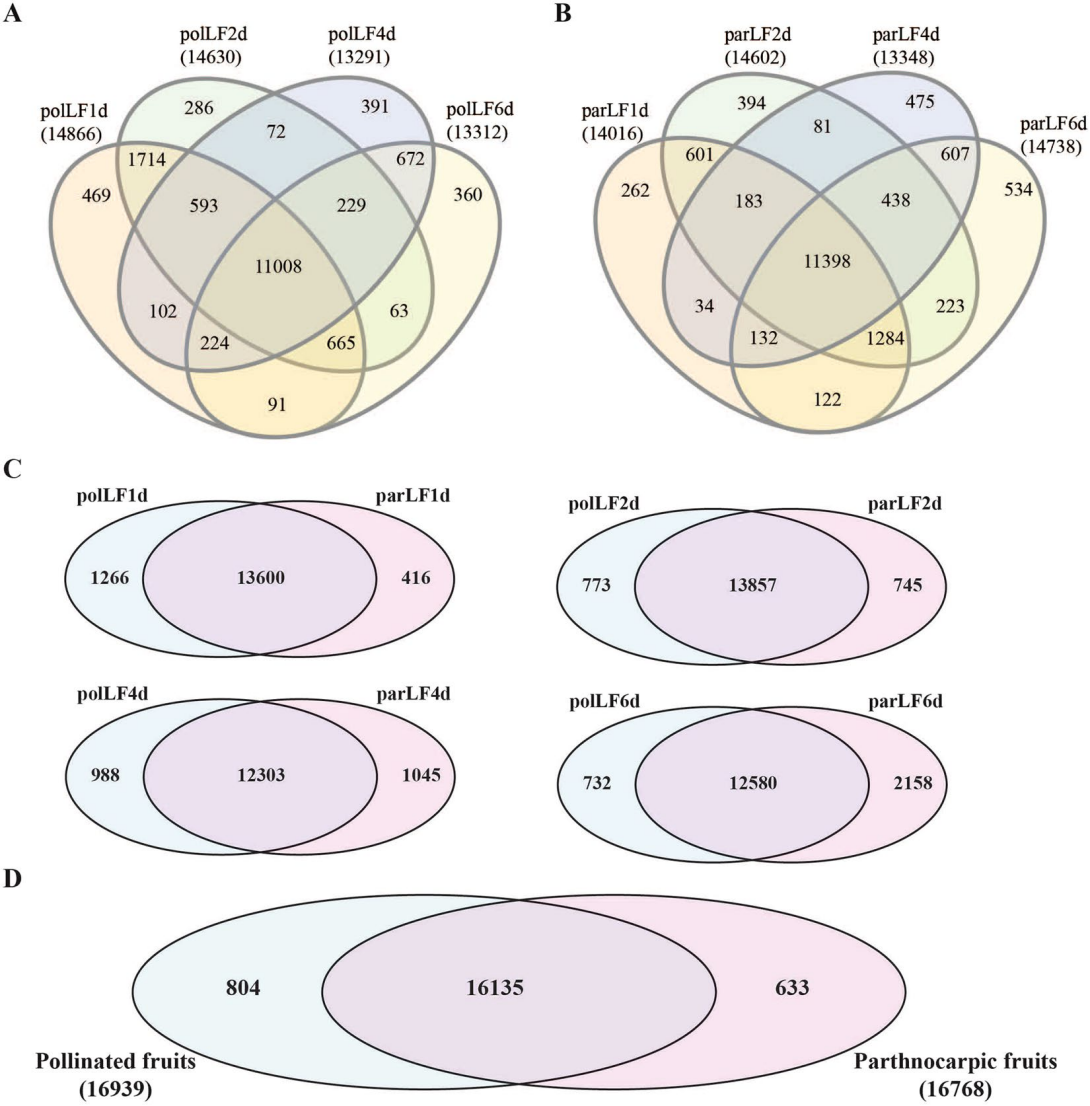
Overview of polLFs and parLFs transcriptomes. (**A**) and (**B**) are the Venn diagrams of commonly/specifically identified genes in polLFs and parLFs, respectively. (**C**) Comparisons of identified genes in polLFs and parLFs at different stages. (**D**) Overall of the identified genes in polLFs and parLFs.

To study the gene expression changes in pollinated (polLFs) and parthenocarpic (parLFs) ‘Hexiachuan’ litchi fruits during early ovary development, we obtained the polLFs ovaries after flower pollination for 1 d (polLF1d), 2 d (polLF2d), 4 d (polLF4d) and 6 d (polLF6d) and the parLFs ovaries after anthesis for 1 d (parLF1d), 2 d (parLF2d), 4 d (parLF4d) and 6 d (parLF6d). Then, the total RNA of each sample was extracted by TRIzol reagent and was sequenced by the Illumina HiSeq 2000 platform. Initially, the transcriptome sequencing generated ~48.95 million raw reads and ~48.73 million clean reads for all samples (Table 1). Then, the clean reads were aligned to the litchi genome sequence for each sample, resulting in 81.20% to 84.56% of clean reads with no more than a 3-base mismatch. To profile litchi gene expression, we counted the number of clean reads aligned to litchi gene sequences (http://litchidb.genomics.cn/, 65,076 sequences) and performed normalization using the RPKM (reads per kilobase per million reads) method. After lowly expressed genes (<5 RPKM) were filtered, we identified 17,572 genes (27.00% of all litchi genes) in all samples. In polLFs, a total of 11,008 genes were commonly identified while 469, 286, 391 and 360 genes were exclusively identified in poLF1d, polLF2d, polLF4d and polLF6d, respectively (Fig. 1A). In parLFs, a total of 11,398 genes were commonly identified while 262, 394, 475 and 534 genes were exclusively identified in parLF1d, parLF2d, parLF4d and parLF6d, respectively (Fig. 1B). Then, we compared genes identified in polLFs and parLFs and 12,303~13,857 genes were commonly identified in polLFs and parLFs at different time stages (Fig. 1C). It is interesting that 469 genes were exclusively identified in parLFs at more than one time point, 2 of which were identified at all time points (Litchi_GLEAN_10009945 and Litchi_GLEAN_10010833, corresponding to ACA12 and ZFP6, respectively). In summary, 16,135 genes were commonly identified in polLFs and parLFs while 804 and 633 genes were identified only in polLFs and parLFs, respectively (Fig. 1D). The commonly and specifically identified genes in polLFs and parLFs allow us to investigate the genes involved in the early development of pollinated and parthenocarpic litchi fruits. The distribution of gene expression (Fig. S1) revealed that 63.20%~66.56% of the total identified genes were between 10 to 100 RPKM, but we still identified 0.36%~0.76% of the total detected genes were more than 1,000 RPKM, and the key genes of this subset maybe involved in early litchi fruit development.

**Table 1:** Overview of litchi fruit transcriptome.

Sample	Raw_reads	Clean_reads	Aligned_to_genome	Ratio	Aligned_to_gene	Ratio	Gene_number
parLF1d	6,327,873	6,299,667	5,208,095	82.67%	3,982,166	63.21%	14,016
parLF2d	5,896,022	5,870,575	4,859,366	82.77%	3,644,864	62.09%	14,602
parLF4d	6,003,645	5,980,851	5,057,414	84.56%	3,700,270	61.87%	13,348
parLF6d	6,331,485	6,305,596	5,221,172	82.80%	4,103,675	65.08%	14,738
polLF1d	6,015,576	5,986,046	4,860,544	81.20%	3,357,589	56.09%	14,866
polLF2d	6,101,442	6,074,300	5,019,468	82.63%	3,789,257	62.38%	14,630
polLF4d	6,197,368	6,170,686	5,132,364	83.17%	3,940,139	63.85%	13,291
polLF6d	6,075,887	6,046,939	4,937,553	81.65%	3,354,528	55.47%	13,312

### DEGs in pollinated and parthenocarpic litchi fruits

**Figure 2:**
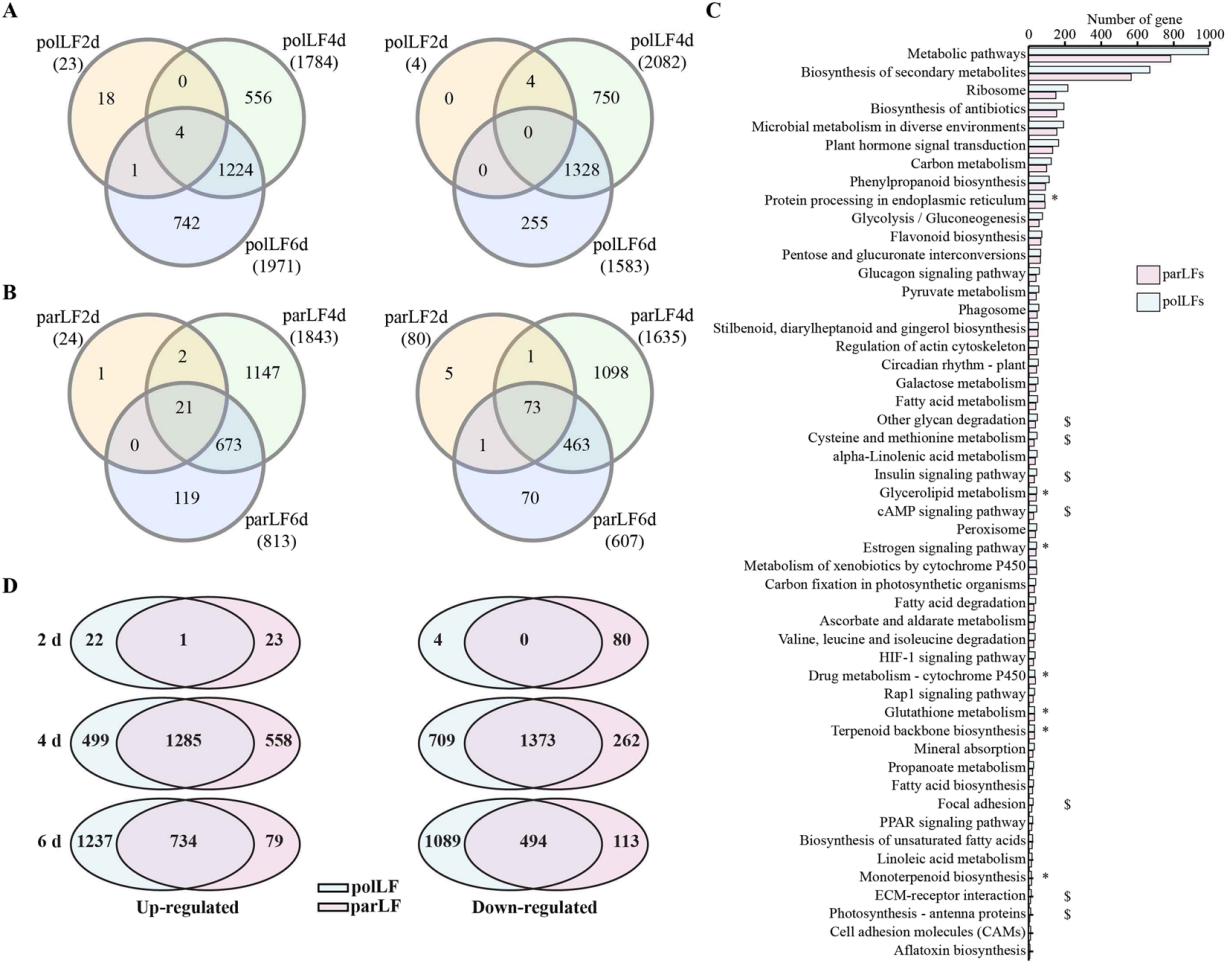
DEGs identified in polLFs and parLFs. edgeR was used to identify the DEGs with strict criterial (log2FC >1 or log2FC <−1, FDR <0.05) in polLFs (**A**) and parLFs (**B**). KEGG pathway analysis (**C**) revealed most of the pathways were shared by DEGs in polLFs and parLFs. * and $ represent the pathways identified with significant enrichment by the DEGs only in polLFs and parLFs, respectively (**D**) Venn diragrams of DEGs identified in polLFs and parLFs at different developmental stages. Left panel: up-regulated genes; right panel: down-regulated genes.

To investigate litchi fruit development associated genes, we performed differential gene expression analysis in polLFs and parLFs using edgeR^33^ and employed a strict criteria (log_2_ fold change (log2FC) > 1 or log2FC < −1, FDR < 0.05). In polLFs we identified 4,864 DEGs (Supplementary Dataset) after 2 d of flower pollination compared to polLF1d, of which 27 (23 up-regulated and 4 down-regulated), 3,866 (1,784 up-regulated and 2,082 down-regulated) and 3,554 (1971 up-regulated, 1583 down-regulated) were distributed in polLF2d, polLF4d and polLF6d, respectively (Fig. 2A). Venn diagrams revealed only 4 genes were commonly up-regulated in all polLFs while polLF4d and polLF6d shared a large set of dysregulated genes (1,224 up-regulated and 1,328 down-regulated). The 18 up-regulated exclusively in polLF2d include heat stress associated genes, such as ERDJ3A, a co-molecular chaperone that is required for the normal growth of pollen tubes under high-temperature stress^34^, heat shock proteins (HSPs, e.g., HPBP1, HS25P, HSP41 and HSP16), DnaJ proteins (e.g., DJB13, DNJH, DNJB6 and DNJ3) and CLPB1, a molecular chaperone required for long-term acquired thermotolerance (LAT) in plants^35^. It is clear that most of the DEGs were identified in polLF4d and polLF6d, indicating that litchi fruit development is affected by the pollination after two or three days, which is consistent with our long-term observation research^30^.

Next, using the same method we identified 3,672 DEGs (Supplementary Dataset) in parLFs compared to parLF1d, of which 104 (24 up-regulated and 80 down-regulated), 3,478 (1,843 up-regulated and 1,635 down-regulated) and 1420 (813 up-regulated and 607 down-regulated) were distributed in parLF2d, parLF4d and parLF6d, respectively. Like polLFs, the difference between parLF1d and parLF2d is small. Venn diagrams (Fig. 2B) showed that 21 up-regulated and 73 down-regulated genes were shared by parLF2d, parLF4d and parLF6d. The commonly up-regulated genes include two genes encoding DnaJ proteins (DNAJ8 and DNAJ11) and one gene encoding MYB108 transcription factor (TF). Unlike polLFs, genes encoding heat shock proteins (HSPs) were commonly down-regulated in parLFs, including HS174, HS17C, HS21C and HSP12. It is interesting that 2,245 out of 3,672 DEGs (61.14%) were exclusively identified in parLF4d, indicating the great changes that occurred in the parLFs of anthesis for 3–5 days.

KEGG pathway enrichment analysis (Fig. 2C and Table S1) showed that most of the significant pathways were shared by DEGs in polLFs and parLFs. Six pathways involved more than 100 DEGs in polLFs and parLFs, including ‘Metabolic pathways’ (q-value = 7.57e-14 in polLFs and parLFs), ‘Biosynthesis of secondary metabolites’ (q-value = 7.57e-14 in polLFs and parLFs), ‘Microbial metabolism in diverse environments’ (q-value = 8.92e-08 in polLFs and 2.08e-07 in parLFs), ‘Biosynthesis of antibiotics’ (q-value = 1.53e-11 in polLFs and 7.54e-10 in parLFs), ‘Ribosome’ (q-value = 7.57e-14 in polLFs and 7.44e-14 in parLFs), and ‘Plant hormone signal transduction’ (q-value = 2.21e-10 in polLFs and 4.13e-09 in parLFs). Also, several pathways were exclusively enriched by the DEGs in polLFs or parLFs. For example, ‘Protein processing in endoplasmic reticulum’ (q-value = 0.18 in polLFs and 1.93e-06 in parLFs) was only found in parLFs while ‘cAMP signalling pathway’ (q-value = 1.28e-05 in polLFs and 0.26 in parLFs) was only found in polLFs. It is interesting that most of the significant GO terms annotated by the DEGs are different between polLFs (Table S2) and parLFs (Table S3), especially in the biological process category. In polLFs 19 and 13 DEGs were identified as involved in the processes of ‘GO:0006098 ~ pentose-phosphate shunt’ (q-value = 2.1e-05) and ‘GO:0080129~proteasome core complex assembly’ (q-value = 0.005), respectively. In parLFs we found 21 DEGs involved in the biological process of ‘GO:0050896~response to stimulus’ (q-value = 0.003). These results indicate that different pathways are triggered in polLFs and parLFs in the early developmental stage.

### Litchi fruit development-associated genes

A comparison of DEGs in polLFs and parLFs revealed a large set of shared DEGs (Fig. 2D), which might include litchi fruit development associated genes. Most of the shared DEGs were identified in fruits after 4 days of development, so here we focused on these genes and annotated them into several known gene groups that were functional in plant development (Table 2), including ABA-related, auxin-related, ethylene-related, GA-related, HSP, histone, RP, TF, ZFP-associated genes. In each category, shared DEGs were a large part of the total DEGs in polLFs and parLFs. For example, 3 ABA-related genes (PYL3, ABF2 and NCED1) were up-regulated in both polLFs and parLFs; 16/21 auxin-related genes were down-regulated, including auxin-response proteins (IAA11, IAA13, IAA27, IAA29) and auxin transporter-like protein LAX5. Some GA-related genes were down-regulated in both polLFs and parLFs, such as GA20OX1 (Gibberellin 20 oxidase 1), GASA3 (Gibberellin-regulated protein 3) and GAI (DELLA protein). It is notable that 44 HSP, 25 histone and 113 RP genes were down-regulated in both polLFs and parLFs. Their down-regulation indicates that new pathways are switched on in litchi fruit development. KEGG pathway analysis (Fig. 2C and Table S1) revealed that most of the pathways were shared by polLFs and parLFs, and in these pathways, we found 109 shared DEGs in ‘Plant hormone signal transduction’ and 87 in ‘Carbon metabolism’. GO analysis of the shared DEGs in polLFs and parLFs revealed 285 DEGs that are involved in biological processes including ‘GO:0055114 ~ oxidation-reduction process’ (q-value = 0.036564), 37 DEGs of ‘GO:0022625 ~ cytosolic large ribosomal subunit’ (q-value = 9.92e-12) and 24 DEGs of ‘GO:0022627 ~ cytosolic small ribosomal subunit’ (q-value = 1.22e-09).

**Table 2:** Genes commonly up- and down-regualted in polLF and parLF during the early fruit developmental process.

Gene_class	Total_in_litchi^a^	polLF^b^	parLF^c^	Common^d^	parLF_only^e^	parLF vs. polLF^f^
abscisic acid related	57	4/1	4/0	3/0	3/0	2/1
auxin related	367	9/20	8/17	5/16	5/1	7/3
ethylene associated	279	42/11	35/9	28/7	13/2	7/10
gibberellin associated	71	14/4	11/4	7/4	4/0	1/1
heat shock protein	149	8/45	4/50	2/44	2/47	2/17
histone	321	5/31	3/27	2/25	1/4	0/1
ribosomal protein	993	8/177	7/126	4/113	4/22	1/1
transcription factor	1604	120/82	97/60	81/50	27/13	19/23
zinc finger protien associated	680	31/19	23/17	18/9	5/8	8/4

^a^Total number of genes annotated in litchi genome.

^b^Number of DEGs in polLFs, up-regulated/down-regulated.

^c^Number of DEGs in parLFs, up-regulated/down-regulated.

^d^Number of DEGs in both polLFs and parLFs, up-regulated/down-regulated.

^e^Number of DEGs exclusively in parLFs, up-regulated/down-regulated.

^f^Number of DEGs in parLFs compared to polLFs, up-regulated/down-regulated.

### Litchi parthenocarpy-associated genes

To investigate parthenocarpy-associated genes in litchi, we first analysed the 633 genes that were exclusively identified in parLFs (Fig. 1D), of which 140, 216, 173 and 241 were distributed in parLF1d, parLF2d, parLF4d and parLF6d, respectively. These numbers are smaller than the numbers of exclusively identified genes in parLFs shown in Fig. 1C because some special genes that were identified in parLFs at one stage were found at another stage in polLFs. These 633 parLFs genes include 5 auxin-related genes encoding AX6B, AXX15 and IAA30, 6 RP genes (RS23, RS8, RL2, RK3B, RL10 and RL192) and 25 TF genes (2 heat stress TF C-1, 8 MYB, 2 WRKY, 5 bHLH, and 8 other TFs). Next, we analyzed DEGs specifically identified in parLFs (Fig. 2D). In total, 660 up-regulated and 455 down-regulated genes were specifically identified in parLFs at one or more developmental stages, including 3 ABA, 6 auxin, 15 ethylene, 4 GA, 49 HSP, 5 histone, 26 RP, 40 TF and 13 ZFP-related genes (Table 2). It is interesting that 13 ethylene-related genes were up-regulated, including 1A1C (1-aminocyclopropane-1-carboxylate synthase) and some ethylene-responsive TFs, while 47 HSP and 22 RP genes were down-regulated only in parLFs. Further experiments are required to explore the functions of HSP and RP genes in parthenocarpic litchi fruit development.

**Figure 3:**
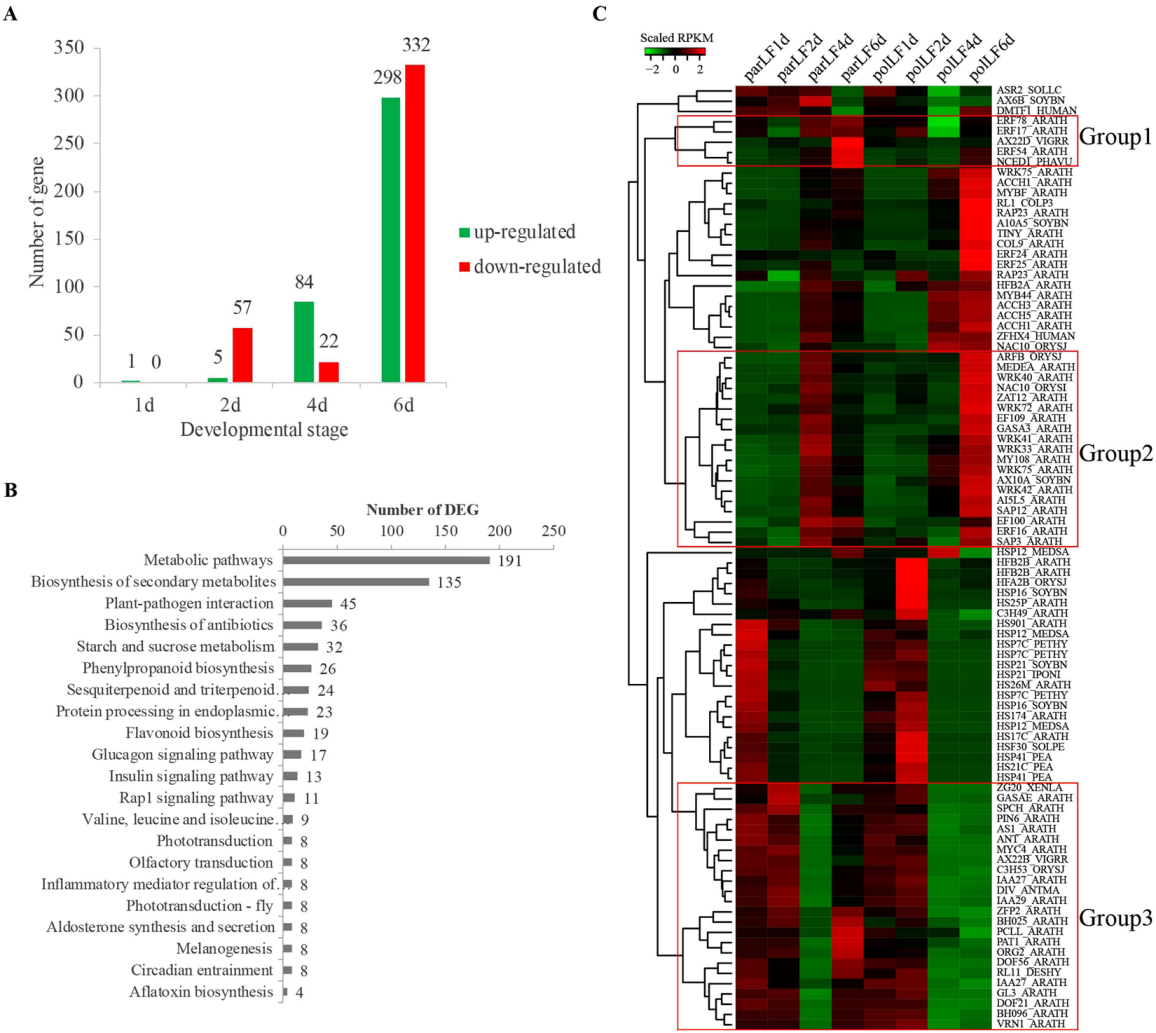
Identification of DEGs in parLFs relative to polLFs at different developmental stages. (**A**) Number of DEGs identified in parLFs compared to polLFs at different developmental stages. (**B**) KEGG pathway enrichment by the DEGs identified in parLFs compared to polLFs. (**C**) Heat map of DEGs associated with ABA, auxin, ethylene, GA, HSP, histone, RP, TF and ZFP identified in polLFs and parLFs.

edgeR^33^ identified 768 DEGs in parLFs compared to polLFs at different developmental stages, of which 1, 62, 106 and 630 distributed into 1 day, 2 days, 4 days and 6 days, respectively (Fig. 3A and Supplementary Dataset). It is interesting that the number of DEGs increased along with fruit development. KEGG pathway analysis (Fig. 3B) showed that the top three pathways involving these DEGs were ‘Metabolic pathways’ (q-value = 5.57e-14), ‘Biosynthesis of secondary metabolites’ (q-value = 5.57e-14) and ‘Plant-pathogen interaction’ (q-value = 6.08e-03). BGLU24 (Beta-glucosidase 24) is the only up-regulated gene in parLF1d compared to polLF1d (log2FC = 6.47, FDR = 1.68e-08). Notably, down-regulated genes in parLF2d contained a large set of heat stress-related genes, including GOLS1 (galactinol synthase 1), a heat shock factor target gene^36^, 5 genes encoding DnaJ proteins, 17 genes encoding HSP and 5 genes encoding other heat stress related proteins (e.g., heat stress TFs). In parLF4d we identified 6 up-regulated genes encoding different ethylene-responsive TFs (e.g., 1 A, ERF16, ERF17, ERF54); however, these genes were down-regulated in parLF6d. In summary, we identified 3, 10, 17, 2, 19, 1, 2, 42 and 12 DEGs related to ABA, auxin, ethylene, HSP, histone, RP, TF and ZFP, respectively (Table 2). A heat map of these DEGs (Fig. 3C) revealed some groups of DEGs that were of interest due to their inconsistent expression patterns in polLFs and parLFs. The first group contains five genes (ERF17, ERF78, AX22D, ERF54 and NCED1) associated with auxin, ethylene and ABA that were significantly up-regulated in parLF6d, and among them, ERF17 and ERF78 were abundant in parLF4d, but were lowly detected in polLF4d. The second group contains 19 genes associated with auxin, GA (e.g., GASA3), ethylene (e.g., EF100, EF109 and ERF16), TF (e.g., MY108, NAC10, WRK33, WRK41 and WRK72) and ZFP (e.g., ZAT12) that were highly expressed in parLF4d; however, their expression significantly decreased in parLF6d, and increased in polLFs. The third group contains 24 genes whose expression was decreased in polLFs but increased in parLFs, especially in parLF6d, and it includes GASAE (GASA14, gibberellin-regulated protein 14). In addition, the expression levels of HSP-related DEGs (mainly between group2 and group3) were quite similar in polLFs and parLFs, but were different between parLFs and polLFs. HSP-related genes were down-regulated in parLF2d compared to polLF2d, indicating that pollination might be the reason for the up-regulation of HSP in polLFs and that HSPs probably function in litchi fruit development.

### Identification of differentially expressed proteins in polLFs and parLFs using iTRAQ

**Figure 4:**
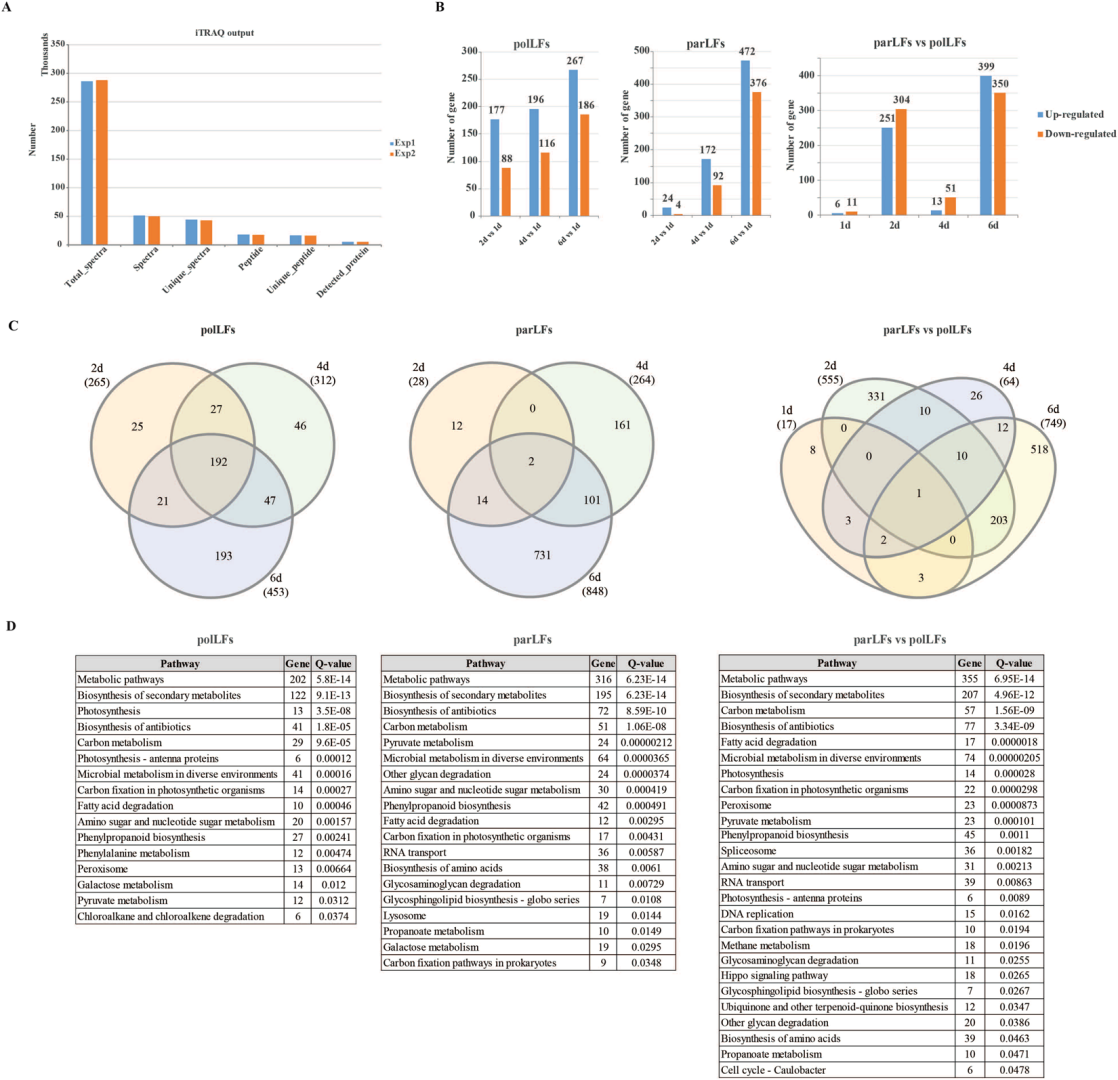
iTRAQ proteomics analysis of polLFs and parLFs. (**A**) Overview features of of two individual iTRAQ experiments. (**B**) Differentially expressed proteins identified. Left: DEPs in polLFs (relative to polLF1d); middle: DEPs in parLFs (relative to parLF1d); right: DEPs in parLFs (relative to polLFs) at different stages. (**C**) Venn diagrams of DEPs. Left: DEPs in polLFs (relative to polLF1d); middle: DEPs in parLFs (relative to parLF1d); right: DEPs in parLFs (relative to polLFs) at different stages. (**D**) Significant (*q* < 0.05) KEGG pathways of DEPs identified in polLFs and parLFs.

Proteomics is a technology that can be used to study the large-scale of structure and function of proteins in complex biological samples^20, 37^. In this study, we applied iTRAQ, which is a relative quantitation proteomics method based on covalent labelling of the N-terminus and side chain amines of peptides from protein digestions with tags of varying mass, to study the protein expression changes in polLFs and parLFs during the early developmental process (1 d, 2 d, 4 d and 6 d). Initially, we obtained 286,153 and 287,769 spectra in two biological individual experiments and other features, such as peptides, unique peptides and detected proteins can be seen in Fig. 4A. In total, 5,814 litchi proteins (out of 65,706) were identified, of which 1,529 were differentially expressed in polLFs and parLFs fruit development (Fig. 4B and Supplementary Dataset). We first investigated the 551 differentially expressed proteins (DEPs) in polLFs (relative to polLF1d), of which 265, 312 and 453 were distributed in polLF2d, polLF4d and polLF6d, respectively (Fig. 4B). A total of 192 DEPs were shared while 25, 46 and 193 DEPs were exclusively identified in polFL2d, polLF4d and polLF6d, respectively (Fig. 4C). KEGG pathway analysis of the DEPs in polLFs (Fig. 4D, left panel) showed that they were mainly involved in biological processes including ‘Metabolic pathways’ (q-value = 5.9e-14) and ‘Biosynthesis of secondary metabolites’ (q-value = 9.1e-13), which is consistent with our previously described transcriptome results. However, not that many DEPs in polLFs were identified in those nine important protein groups (Table 3). We identified only one auxin-related protein (AB19B) that was down-regulated and four histone proteins (H1, H4, H32, KAT5) that were up-regulated in polLFs. The top five up-regulated and down-regulated proteins in polLFs at different developmental stages (Table 4) showed that LEC_PARPC (mannose/glucose-specific lectin) was up-regulated during the developmental process in polLFs and that two proteins (PAL1_PRUAV and PALY_POPTR) which are key enzymes of plant metabolism, catalysing the first reaction in the biosynthesis from L-phenylalanine for a wide variety of natural products based on the phenylpropane skeleton^38^, were down-regulated. Next, we annotated the 1,021 DEPs (Supplementary Dataset) in parLFs into the nine groups (Table 3) and found more dysregulated proteins. For example, the dysregulated proteins included 1 up-regulated (AIR12) and 4 down-regulated (ABCB19, ARF6, BIG, AXR4) auxin-related proteins; 3 up-regulated HSPs (HSP22, HSP7M, HSP70); and 9 up-regulated and 7 down-regulated RPs. KEGG analysis (Fig. 4D, middle panel) showed that the DEPs of parLFs were also involved in biological processes, such as ‘Metabolic pathways’ (q-value = 6.23e-14), ‘Biosynthesis of secondary metabolites’ (q-value = 6.23e-14) and ‘Carbon metabolism’ (q-value = 1.06e-8). More pathways were identified from the DEPs of parLFs, such as ‘Lysosome’ (q-value = 0.014) and ‘Glycosaminoglycan degradation’ (q-value = 0.007). No proteins were shared by the top five up-/down-regulated proteins of parLFs at different developmental stages (Table 4).

**Table 3:** DEPs in polLFs and parLFs during the early developmental process.

Gene_class	Total_in_litchi^a^	polLF^b^	parLF^c^	Common^d^	parLF_only^e^	parLF vs. polLF^f^
abscisic acid related	57	0/0	0/0	0/0	0/0	0/0
auxin related	367	0/1	1/4	0/1	1/3	2/0
ethylene associated	279	1/1	1/2	1/1	0/1	1/2
gibberellin associated	71	0/0	0/0	0/0	0/0	0/0
heat shock protein	149	1/0	3/0	0/0	3/0	0/1
histone	321	4/1	5/0	2/0	3/0	5/4
ribosomal protein	993	2/4	9/7	0/0	9/7	10/16
transcription factor	1604	2/3	1/3	0/0	1/3	4/2
zinc finger protien associated	680	1/2	1/4	0/2	1/3	2/3

^a^Total number of genes annotated in litchi genome.

^b^Number of DEPs in polLFs, up-regulated/down-regulated.

^c^Number of DEPs in parLFs, up-regulated/down-regulated.

^d^Number of DEPs in both polLFs and parLFs, up-regulated/down-regulated.

^e^Number of DEPs exclusively in parLFs, up-regulated/down-regulated.

^f^Number of DEPs in parLFs compared to polLFs, up-regulated/down-regulated.

**Table 4:** Top five up-regulated and down-regulated proteinds identified in polLFs and parLFs by iTRAQ.

polLF2d_vs_polLF1d		polLF4d_vs_polLF1d		polLF6d_vs_polLF1d		parLF2d_vs_parLF1d^a^		parLF4d_vs_parLF1d		parLF6d_vs_parLF1d	
pro_id	ratio	pro_id	ratio	pro_id	ratio	pro_id	ratio	pro_id	ratio	pro_id	ratio
**Up-regulated**											
LEC_PARPC	8.86	ACCH1_ARATH	5.80	T23E18.8	25.27	PRP1_ARATH	2.44	GLYG3_SOYBN	5.33	LAC14_ARATH	4.90
RETOL_ARATH	7.53	HA22K_ARATH	5.78	LEC_PARPC	11.70	VPE_CITSI	2.42	EMJ07221.1	4.70	MIRA_SYNDU	3.96
GLYG3_SOYBN	7.26	LEC_PARPC	5.51	GLYG3_SOYBN	9.44	CYT1_ACTDE	2.33	ACCH1_ARATH	4.64	Y5258_ARATH	3.69
CHIB_TOBAC	7.23	PER4_VITVI	4.81	RETOL_ARATH	7.34	T23E18.8	2.23	GDL76_ARATH	4.27	CLPP4_ARATH	3.58
TLP1_PRUPE	7.18	EMJ07221.1	4.55	LEC_PARPC	7.01	Y4744_ARATH	1.93	E13B_HEVBR	3.63	LEC_PARPC	3.41
**Down-regulated**											
C82G1_ARATH	0.24	PALY_POPTR	0.23	PALY_POPTR	0.24	T23E18.8	0.12	PALY_POPTR	0.19	HA22K_ARATH	0.12
SSL12_ARATH	0.28	PAL1_PRUAV	0.24	CHS1_CAMSI	0.25	HA22K_ARATH	0.17	PAL1_PRUAV	0.21	T23E18.8	0.16
PAL1_PRUAV	0.30	SSL12_ARATH	0.29	PAL1_PRUAV	0.26	ADHX_ARATH	0.60	LAR_DESUN	0.28	C82G1_ARATH	0.24
LAR_DESUN	0.31	C82G1_ARATH	0.29	LAC14_ARATH	0.27	CFTSY_ARATH	0.63	ELIP1_ARATH	0.29	CBF5_ASPFU	0.27
PALY_POPTR	0.31	LAR_DESUN	0.30	AGO4_ARATH	0.29			AROF_ARATH	0.29	PR35B_ARATH	0.29

^a^Only four down-regulated proteins identified in parLF2d compared to parLF1d.

We compared the DEPs identified in polLFs and parLFs and found that 305 were shared, of which 3, 157 and 145 were distributed in the fruits at 2 days, 4 days and 6 days, respectively (Fig. S2). Among them, we found 1, 2, 2, and 2 DEPs associated with auxin, ethylene, histone, and ZFP, respectively (Table 3). KEGG pathway analysis (Table S4) showed that some litchi fruit development-associated pathways might involved the common DEPs in polLFs and parLFs, such as ‘Amino sugar and nucleotide sugar metabolism’^39^ (q-value = 3.13e-3) and ‘Photosynthesis’^40^ (q-value = 1.66e-3). Notably, in the top five up-/down-regulated proteins (Table 4) we found some shared proteins that might be associated with litchi fruit development. For example, 2 up-regulated proteins (ACCH1 and EMJ07221.1) and 3 down-regulated proteins (PALY_POPTR, PAL1_PRUAV, LAR_DESUN) were common to polLF4d and parLF4d; LEC_PARPC was up-regulated in polLF6d and parLF6d.

### Parthenocarpy-associated proteins in litchi

Like the transcriptome analysis, we identified 526 DEPs exclusively in parLFs, of which 25 (21 up-regulated and 4 down-regulated), 107 (60 up-regulated and 47 down-regulated) and 502 (174 up-regulated and 328 down-regulated) were distributed in parLF2d, parLF4d and parLF6d, respectively (Fig. S2). Annotation of these proteins showed all the DEPs related with HSP, RP and TF were exclusive to parLFs (Table 3). In addition, 3/4 auxin-related DEPs (excluding AB19B) were identified only in parLFs. KEGG pathway analysis (Table S5) showed 130, 28 and 22 parLFs special DEPs involved in the pathways of ‘Metabolic pathways’ (q-value = 3.29E-11), ‘Biosynthesis of antibiotics’ (q-value = 3.12E-02) and ‘Carbon metabolism’ (q-value = 4.88E-03), respectively.

**Figure 5:**
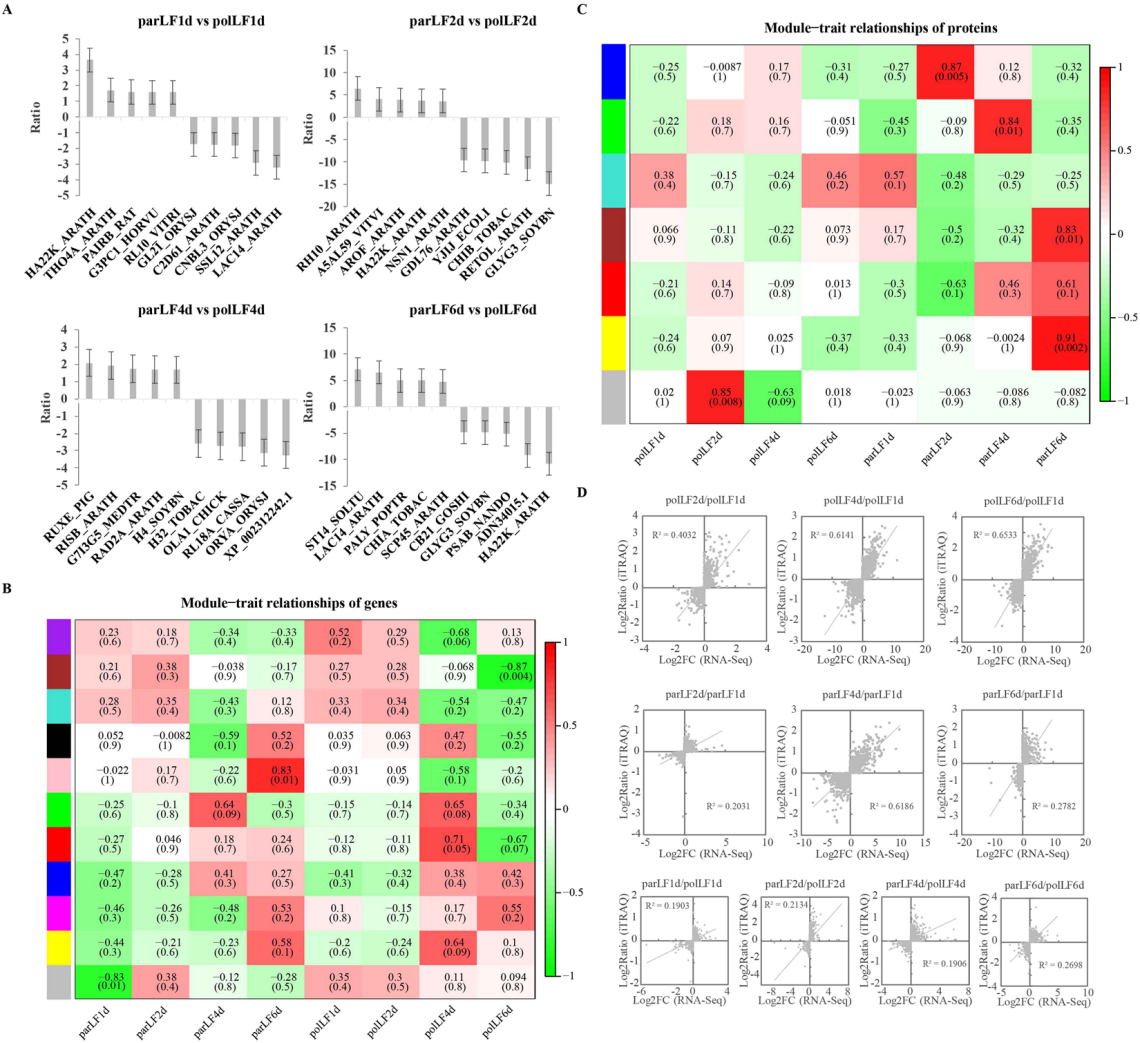
Comparison of DEGs and DEPs identified in this study. (**A**) Top 5 up-/down-regulated proteins in parLFs compared to polLFs at different time stages. Error bars stand for the standard division. (**B**) Module-trait relationships of co-expressed genes in polLFs and parLFs. (**C**) Module-trait relationships of co-expressed proteins in polLFs and parLFs. (**D**) Scatter plots of DEGs/DEPs in polLFs and parLFs identified transcriptome sequencing and iTRAQ technologies.

Compared to polLFs, we identified 17 (6 up-regulated and 11 down-regulated), 555 (251 up-regulated and 304 down-regulated), 64 (13 up-regulated and 51 down-regulated) and 749 (399 up-regulated and 350 down-regulated) DEPs in parLFs at 1 day, 2 days, 4 days and 6 days, respectively (Fig. 4B and Supplementary Dataset). A Venn diagram (Fig. 4C, right panel) showed only one protein (HVA22K) was dysregulated in parLFs compared to polLFs during early development. Interestingly, HVA22K was up-regulated in parLF1d and parLF2d, but it was down-regulated in parLF4d and parLF6d (Supplementary Dataset). The overexpression of plant HVA22 can inhibit the formation of the large digestive vacuoles induced by GA and promote GA-induced programmed cell death^41^. Although no GA-related proteins were identified in this study, the down-regulation of HVA22 homologue protein indicates a high level of GA in parLFs. Table 3 showed that 2, 3, 1, 9, 26, 6 and 5 DEPs in parLFs and polLFs are associated with auxin, ethylene, HSP, histone, RP, TF and ZPF, respectively. Among the TFs, we found that HBP-1a TF, which binds to the hexamer motif 5′-ACGTCA-3′ of histone gene promoters^42^, was up-regulated in parLF6d compared to polLF6d, and that 2 DIVARICATA TFs, which controls the flower shape in *Antirrhinum majus* ^43^, were up-regulated. KEGG pathway analysis (Table S6) showed that ‘Metabolic pathways’ is the most significant pathway among all those involving the DEPs between parLFs and polLFs. However, we found 24 DEPs that function in ‘spliceosome’ (q-value = 3.35e-4) in parLF2d and some that function in energy metabolism pathways in parLF6d. We showed the top five up-/down-regulated proteins in parLFs compared to polLFs at different developmental stages (Fig. 5A). Unlike the findings in our transcriptome data, most of the top 5 dysregulated proteins were different from one stage to another. HA22K_ARATH (HVA22K) is the most up-regulated protein and LAC14 is the most down-regulated protein in parLF1d, indicating that their change might be a response to the flower pollination and that they probably function in the preparation for litchi parthenocarpy. GLYG3_SOYBN (GY3, glycinin G3), a major protein stored in seeds^44^, is down-regulated in parLF2d compared to polLF2d. In addition, its expression in parLF6d is also lower than its expression in polLF6d (Supplementary Dataset). It is interesting that a plant defensive protein ST14_SOLTU (STS14) was expressed more in parLF6d than polLF6d, but this protein might function in the protection of ovary development without pollination^45^.

### Co-expressed genes/proteins by WGCNA

To understand the co-expression relationship between genes/proteins in parLFs and polLFs, we performed weighted gene co-expression network analysis (WGCNA)^46^ using both transcriptome and iTRAQ results. Using the criterial (Pearson *r* ≥ 0.8 or *r* ≤ −0.8, *p* ≤ 0.05) we found 2 modules (brown and pink) of genes (Fig. 5B) and 5 modules (blue, green, brown, yellow and grey) of proteins of interest (Fig. 5C). The brown and pink modules in the transcriptome data contained 1,801 and 514 genes, respectively (Supplementary Dataset). KEGG pathway analysis revealed that the top 2 pathways involving the co-expressed genes of both the brown and pink modules were ‘Metabolic pathway’ and ‘Biosynthesis of secondary metabolites’ (Table S7). We also found 21 genes from the pink module that were involved in the pathway of ‘Sesquiterpenoid and triterpenoid biosynthesis’ (q-value = 1.36e-10), which is important for ABA biosynthesis^47^. Next, we analysed the co-expressed proteins. In total, five modules with 2,642 proteins were identified, of which 992, 806, 323, 33 and 489 were distributed in the blue, brown, green, grey and yellow modules, respectively (Supplementary Dataset). Interestingly, the significant pathways, such as ‘Metabolic pathway’ and ‘Biosynthesis of secondary metabolites’ also involved the co-expressed proteins in all five modules (Table S8).

A comparison of the WGCNA results identified 333 genes/proteins that were co-expressed (Supplementary Dataset) on both RNA and protein levels. In detail, we divided them into several groups and the two largest groups contained 109 (module colour: RNA-Seq - brown, iTRAQ - blue) and 88 (module colour: RNA-Seq - brown, iTRAQ - brown) genes/proteins. Then, we compared these 333 genes with the DEGs identified in this study, and 50 and 14 were differentially expressed in polLFs and parLFs, respectively. In addition, 12 were dysregulated in parLFs compared to polLFs. Next, we found that these 333 proteins contained 19 and 85 DEPs of polLFs and parLFs, respectively. Compared to polLFs, 84/333 proteins were differentially expressed in parLFs at all developmental stages. These results support the fact that the co-expressed genes/proteins might be associated with not only litchi fruit development but also litchi parthenocarpy.

### Cross-validation of transcriptomics and proteomics data

In the current study, we profiled both mRNAs and proteins in polLFs and parLFs using deep sequencing and iTRAQ technologies, which enabled us to use the output of one experiment to support the other one^48–50^. A cross comparison of the identified DEGs and DEPs showed that 357 of them were dysregulated on both RNA and protein levels (Supplementary Dataset). During the development of polLFs, we found that 281 DEGs/DEPs were consistent, of which 1 (1 up-regulated), 199 (133 up-regulated and 66 down-regulated) and 224 (152 up-regulated and 72 down-regulated) were distributed into polLF2d, polLF4d and polLF6d, respectively. On the other hand, in parLFs we found 206 consistent DEGs/DEPs, of which 181 (134 up-regulated and 47 down-regulated) and 54 (34 up-regulated and 20 down-regulated) were distributed in parLF4d and parLF6d, respectively. Interestingly, 25 (23 up-regulated and 2 down-regulated) of the consistent DEGs/DEPs were found in both polLFs (polLF4d and polLF6d) and parLFs (parLF4d and parLF6d), and these DEGs/DEPs might be related with litchi fruit development (Table 5). We found that 3/25 of the DEGs/DEPs are from ABC transporter family, including AB13B, AB22G and AB28B, which are related to ABA transport and responses^51^ and are up-regulated in apple fruit early development^52^. In addition, one amidase protein (AMI1), which controls auxin biosynthesis^53^, was down-regulated in both polLFs and parLFs after 2 days of development. Although GY3 was down-regulated in parLFs compared to polLFs, it was up-regulated in polLFs/parLFs during early development.

**Table 5:** Consistent DEGs/DEPs in both polLFs and parLFs.

ID	polLF4d_vs_polLF1d		polLF6d_vs_polLF1d		parLF4d_vs_parLF1d		parLF6d_vs_parLF1d		pro_id	description
	RNA-Seq^a^	iTRAQ^b^	RNA-Seq^a^	iTRAQ^b^	RNA-Seq^a^	iTRAQ^b^	RNA-Seq^a^	iTRAQ^b^		
Litchi_GLEAN_10007037	3.69	2.64	5.38	3.48	3.54	1.77	3.50	1.88	7SB1_SOYBN	Basic 7S globulin
Litchi_GLEAN_10043707	5.78	4.54	7.14	9.44	8.01	5.33	7.48	2.30	GLYG3_SOYBN	Glycinin G3
Litchi_GLEAN_10048452	4.33	3.85	3.97	5.65	6.49	1.96	4.29	1.76	LAC7_ARATH	Laccase-7
Litchi_GLEAN_10018848	2.89	3.44	3.71	5.34	3.46	3.63	2.39	2.41	E13B_HEVBR	Glucan endo-1,3-beta-glucosidase, basic vacuolar isoform
Litchi_GLEAN_10040929	5.86	4.07	4.37	5.06	5.88	3.16	2.98	2.31	PERX_NICSY	Lignin-forming anionic peroxidase
Litchi_GLEAN_10012603	4.69	3.89	4.99	7.34	6.66	3.06	5.11	2.20	RETOL_ARATH	Reticuline oxidase-like protein
Litchi_GLEAN_10057113	3.37	3.74	3.45	5.23	6.27	2.08	3.78	2.21	TLP1_PRUPE	Thaumatin-like protein 1
Litchi_GLEAN_10011351	3.14	3.33	3.80	5.17	4.30	3.15	2.46	2.16	MIRA_SYNDU	Miraculin
Litchi_GLEAN_10063755	4.97	3.77	3.28	7.01	6.35	1.80	1.46	3.41	LEC_PARPC	Mannose/glucose-specific lectin
Litchi_GLEAN_10035130	5.10	3.84	5.50	5.53	8.28	4.27	7.54	1.77	14336_ARATH	14–3–3-like protein GF14 lambda
Litchi_GLEAN_10041708	7.16	3.65	8.48	4.72	5.92	1.77	7.22	1.74	4CLL7_ARATH	4-coumarate–CoA ligase-like 7
Litchi_GLEAN_10007038	6.84	2.26	5.14	6.67	5.47	2.14	4.87	2.16	7SB1_SOYBN	Basic 7S globulin
Litchi_GLEAN_10053354	5.49	4.55	6.45	4.10	5.35	4.70	4.66	2.61	AAP6_ARATH	Amino acid permease 6
Litchi_GLEAN_10060078	4.30	3.55	3.65	4.57	5.56	2.16	4.11	2.68	AASS_ARATH	Alpha-aminoadipic semialdehyde synthase
Litchi_GLEAN_10010547	3.82	2.20	3.01	3.70	4.87	1.59	3.33	2.60	AAT_AQUAE	Aspartate aminotransferase
Litchi_GLEAN_10024158	4.52	2.02	4.06	3.02	3.85	3.16	2.96	1.72	AB28B_ARATH	ABC transporter B family member 28
Litchi_GLEAN_10059709	6.67	1.68	3.64	2.31	3.64	1.61	2.91	1.91	ACA8_ARATH	Calcium-transporting ATPase 8, plasma membrane-type
Litchi_GLEAN_10008840	4.03	2.91	3.33	3.10	3.48	2.48	2.48	1.69	ACBP4_ARATH	Acyl-CoA-binding domain-containing protein 4
Litchi_GLEAN_10001011	3.99	4.81	3.42	4.46	4.32	3.26	2.26	1.98	ACOX1_ARATH	Peroxisomal acyl-coenzyme A oxidase 1
Litchi_GLEAN_10021664	2.93	1.99	2.95	2.50	2.37	2.21	2.04	2.57	ACOX4_ARATH	Acyl-coenzyme A oxidase 4, peroxisomal
Litchi_GLEAN_10063174	3.85	1.97	1.69	2.20	3.10	1.67	1.92	2.63	AGAL_COFAR	Alpha-galactosidase
Litchi_GLEAN_10065085	2.11	1.65	2.41	3.98	3.03	2.93	1.46	1.89	AIM1_ARATH	Peroxisomal fatty acid beta-oxidation multifunctional protein AIM1
Litchi_GLEAN_10034256	2.31	1.94	2.12	1.88	2.39	1.79	1.24	1.54	ALLD3_ACTDE	Allergen Act d 3
Litchi_GLEAN_10032316	−2.57	−2.08	−1.95	−2.39	−2.39	−2.08	−1.27	−2.01	AMI1_ARATH	Amidase 1
Litchi_GLEAN_10008394	−3.51	−2.11	−3.36	−2.46	−3.30	−1.83	−1.28	−2.00	AMO_ARATH	Primary amine oxidase

^a^Fold change of gene expression identified by RNA-Seq.

^b^Ratio of protein expression identified by iTRAQ.

Next, we compared the DEGs/DEPs identified in parLFs compared to polLFs and identified 71 (39 up-regulated and 32 down-regulated) that were consistent on RNA and protein levels (Table S9). Notably, these 71 consistent DEGs/DEPs were all from a comparison of parLF6d to polLF6d. Here, GY3 was also down-regulated in parLF6d compared to polLF6d using transcriptome sequencing and iTRAQ technologies. One of the top five dysregulated proteins (previously described), LEC_PARPC, was also consistent in our analysis. We also found 3 DEGs/DEPs related to glucan endo-1,3-beta-glucosidase (similar to GNS1, HGN1 and At5g56590), whose homologue protein GSN3 is highly expressed during early fruit set in *Prunus persica* ^54^, and another 3 encoding subtilisin-like protease, a developmental regulator that controls the structure and mechanical properties of Arabidopsis seed coat^55^.

To compare the transcriptome and proteome in polLFs and parLFs, we used scatter plots (Fig. 5D) to show the consistent DEGs/DEPs in polLFs and parLFs that were identified by transcriptome sequencing and iTRAQ technologies. The R-square values of consistent DEGs and DEPs in polLF4d and polLF6d (relative to polLF1d) were 0.8473 and 0.7575, respectively. While the R-square values of consistent DEGs/DEPs in parLF4d and parLF6d were 0.8034 and 0.6968, respectively. In parLF6d compared to polLF6d the R-square value of consistent DEGs/DEPs was 0.8013 (Fig. S3). These results revealed excellent agreement between transcriptome sequencing and iTRAQ proteomics anlaysis.

## Discussion

In this study, we analysed the expression of genes and proteins in pollinated and parthenocarpic litchi fruits during different developmental stages using transcriptome sequencing and iTRAQ technologies. Like other studies using both transcriptome sequencing and proteomics^56, 57^, we used these results to support one another in terms of the expression patterns of genes/proteins during development. We analysed the DEGs/DEPs that were mainly found in nine functional groups of genes/proteins in plant: ABA, auxin, ethylene, GA, HSP, histone, RP, TF and ZFP.

Auxin and GA have been shown to function in fruit initiation and development because exogenous hormone treatments of auxin and GA can stimulate parthenocarpy^58^ and their endogenous levels are elevated in ovaries after fertilization^1, 59^. Mutant ARF8 (auxin response factor 8) can stimulate parthenocarpy in both Arabidopsis and tomato^17^. In unpollinated tomato ARF7 is also found at a high level in the ovaries that can form seedless (parthenocarpic) fruits^60^. There are 365 auxin-related proteins in litchi, of which 139 are auxin response factors. In the transcriptome results, we found ARFB, ARFD and ARFE were dysregulated during the developmental process in polLFs and parLFs (Supplementary Dataset). Among them, ARFB was up-regulated in polLF6d and parLF4d. However, compared to polLF6d ARFB mRNA was down-regulated in parLF6d. We also found that ARFF was down-regulated in parLFs on protein level. In addition, we found that some genes encoding auxin-responsive proteins such as IAA27 and IAA29 up-regulated in parLF6d compared to polLF6d. GA has been shown to induce parthenocarpy in citrus^61^, apple^62^ and pear^63^ and three GAs (GA3, GA4 and GA7) have been characterized^64^. Parthenocarpy in tomato mutant (*pat*) was mediated by the mis-regulation of GA20ox1 (Gibberellin 20 oxidase 1) expression^65^, and parthenocarpic fruit growth was induced by overexpressing of the citrus gene CcGA20ox1 in tomato^66^. Consistent with a SlDELLA loss of function, the tomatoes displayed a GA-constitutive response phenotype, including parthenocarpy^67, 68^. In this study, the GA20ox1 gene was up-regulated at 4 days and 6 days after anthesis and the gene (GAI) encoding DELLA protein was down-regulated at 4 days in both parLFs and polLFs (Fig. 3C). In addition, compared to polLFs we found one gene encoding GASAE_ARATH (GASA14) that was highly expressed (>1000 RPKM) and up-regulated in both parLF4d and parLF6d (Fig. 3C). By modulating reactive oxygen species accumulation, GASA14 can regulate leaf expansion and abiotic stress resistance in Arabidopsis^69^. The regulation mechanism of GASA14 in litchi parthenocarpy requires exploration with further experiments.

In the current study, we also studied the expression changes of other gene families, such as ABA, ethylene, HSP, histone, RP, TF and ZFP. Some of them have been shown to be associated with parthenocarpy in other organisms. We found an up-regulated ABA-related gene named NCED1 (9-cis-epoxycarotenoid dioxygenase NCED1, chloroplastic) in parLFs only (Supplementary Dataset), whose homologue protein NCED6 (up-regulated in parLFs but FDR > 0.05) controls ABA biosynthesis in Arabidopsis^70^. In a previous study, we showed the continued decline of ABA after anthesis in both pollinated and parthenocarpic ‘Hexiachuan’ litchi fruits^31, 32^. Therefore, the relationship of NCED1 and NCED6 in litchi parthenocarpy requires exploration with further experiments. In tomato, some of the ethylene-associated genes are down-regulated in transgenic parthenocarpic fruits while some are up-regulated^71^; these result was also found in this study (Table 2). In loquat, two NAC TF genes were found negatively regulated by GA in the fruit setting^23^ and PHOR1 TF and some other TFs are down-regulated in parthenocarpic tomato fruit^71^. We found two NAC genes (Litchi_GLEAN_10027104 and Litchi_GLEAN_10042979) and one NAC protein (Litchi_GLEAN_10023105) that were down-regulated in parLFs compared to polLFs (Supplementary Dataset). We also found another three TF subgroups including bHLH, WRKY and MYB dysregulated in parLF6d and polLF6d. Two MYB TFs (MYB44 and MYB108) were down-regulated in parLFs (Supplementary Dataset); 11 WRKY genes were exclusively down-regulated in parLF6d relative to polLF6d; and unlike the WRKY TFs, 2 bHLH TF genes (bHLH25 and bHLH96) were up-regulated in parLF6d (Supplementary Dataset). A transcriptome study of tomato parthenocarpy mediated by auxin and GA also identified the down-regulation of WRKY TFs and the up-regulation of bHLH TFs^18^. Two studies also reported the up-regulation of histone genes in parthenocarpy tomato^18, 72^. However, we found that three histone genes were up-regulated in parLF4d (compared to polLF4d) but two highly expressed histone genes were down-regulated in parLF6d (compared to polLF6d).

This is the first time that it has been reported that HSP- and ZFP-associated genes might regulate the fruit set of parthenocarpy. HSPs are known to be temperature-related evolutionarily conserved chaperone proteins^73^ and it has been reported that they are be expressed under multiple stresses such as cold, UV light, salt and drought^73^. It is interesting that the down-regulation of HSPs in orthodox seeds is a rapid response to water loss and that their overexpression could be an efficient way to increase tolerance to drought stress^74^. We found that both HSP genes and proteins were down-regulated in parLFs compared to polLFs (Tables 2 and 3), indicating that HSPs might be functional in litchi fruit development and parthenocarpy fruit setting. On both RNA and protein levels we found dysregulated ZFPs in parLFs compared to polLFs (Tables 2 and 3). It is notable that the expression patterns of ZAT12_ARATH, which is encoded by Litchi_GLEAN_10012258, were opposite in parLFs and polLFs (Supplementary Dataset). By interacting with certain COS genes and CFB genes, ZAT12 can increase tolerance to cold, high light, wounding and low-oxygen in plants^75, 76^. Although it is difficult to explain the rapid change of ZAT12 in polLFs and parLFs, its different expression patterns indicate that the ZAT12 pathway might be associated with litchi parthenocarpy.

The fruit set of parthenocarpy is a complicated process, and we know little about it in litchi. The difference between polLFs and parLFs becomes huge for two days after anthesis because more differentially expressed genes and proteins were identified after this time (Figs 2~4). We assume that the decision of litchi fruit set is made ~2 to 4 days after anthesis. The genes and proteins identified in this study provide a valuable resource on parthenocarpy-related gene/protein products, which can benefit the researchers in this field. The findings also contribute to our understanding the global gene/protein changes during fruit development with or without pollination in litchi and improve our understanding of parthenocarpy.

## Methods

### Ethics Statement

No specific permits were required for the described field studies. The location is not privately-owned or protected in any way, and the field studies did not involve endangered or protected species.

### Plant materials and treatment

The ovaries of pollinated and parthenocarpic litchi were collected from the same ‘Hexiachuan’ litchi tree, which was cultivated in the experimental fields of the Institute of Fruit Tree Research, Guangdong Academy of Agricultural Sciences in Maoming, Guangdong of China (21.797314, 111.052974). In brief, during March to April of 2013, the ‘Hexiachuan’ litchi flower season, all the male flowers were removed and the rest were covered with plastic bags. Then, we collected litchi ovaries after the flowers were manually pollinated for 1 day (polLF1d), 2 days (polLF2d), 4 days (polLF4d), and 6 days (polLF6d) and the litchi ovaries from the flowers without pollination (parthenocarpic fruits) at 1 d (parLF1d), 2 d (parLF2d), 4 d (parLF4d), and 6 d (parLF6d). Litchi fruit samples were stored immediately in liquid nitrogen and were transferred to the Institute of Fruit Tree Research (Guangzhou, China) for −80 °C storage. The ovary size of the pollinated and parthenocarpic litchi fruits were measured in a laboratory according to the protocol^77^.

### Isolation of total RNA

Total RNA of pollinated and parthenocarpic litchi fruits was isolated using TRIzol reagent (Invitrogen) according to the manufacturer’s protocol^78^. Initially, 100 mg of litchi fruit was homogenized in 1 mL of TRIzol reagent (Invitrogen) and was centrifuged at 12,000 × g for 10 min at 4 °C. The supernatant was transferred to a new clean tube, incubated at room temperature for 5 min, mixed with 0.2 mL of chloroform and shaken vigorously for 15 s. After an incubation for 3 min at room temperature, the sample was centrifuged at 12,000 × g for 15 min at 4 °C and the aqueous phase was transferred to a new clean tube. Then, 10 µg of RNase-free glycogen (Invitrogen) was added to the tube, followed by the addition of 0.5 mL of 100% isopropanol and incubation for 10 min. After the sample was centrifuged at 12,000 × g for 10 min at 4 °C, the resulting RNA sample was washed and stored at −80 °C.

### cDNA library construction and transcriptome sequencing

Before cDNA library construction, we used NanoDrop1000 (ThermoFisher Scientific) and Agilent Bioanalyser 2100 to evaluate the quality and quantity of total RNA. Then, total RNA (10 µg) was used for cDNA library construction for each sample using TruSeq RNA Library Preparation Kit v2 (Illumina) according to the manufacturer’s protocol. Briefly, after DNase digestion and RNA purification, mRNAs with polyA were isolated using Dynal oligo(dT)-attached magnetic beads (Invitrogen). Then, the mRNAs were chemically fragmented into ~200 bp fragments, and the cleaved mRNAs were synthesized into cDNAs using random hexamer-primers and SuperScript II Reverse Transcriptase (Invitrogen). Second cDNA synthesis was performed using DNA Polymerase I (Invitrogen) and RNase H (Invitrogen). After purification, end-repair and ligation of Illumina sequencing adaptors, the cDNA fragments were gen-purified using a 1.5% Tris-borate-EDTA polyacrylamide gel (Invitrogen) and amplified by PCR. Amplified cDNA libraries were evaluated by Agilent 2100 Bioanalyzer and qRT-PCR. Final cDNA libraries were sequenced with an Illumina HiSeq2000 system in the Beijing Genomics Institute of Shenzhen (BGI-SZ). The following parameters were used for sequencing: insert size, 200 bp; sequencing type, single-end; and read length, 50 bp. The raw files (FASTQ format) can be accessed in the NCBI Sequence Read Archive (SRA) platform (https://trace.ncbi.nlm.nih.gov/Traces/sra/sra.cgi?) under the accession number SRA543489.

### Transcriptome data analysis

Images generated by the sequencer were converted into nucleotide sequences (raw sequencing reads) using a base-calling pipeline (Illumina). Then, the raw reads were quality controlled by FASTQC (http://www.bioinformatics.babraham.ac.uk/projects/fastqc/) and were cleaned by removing low quality reads, contamination reads and reads with adapters using SOAPnuke (http://www.seq500.com/uploadfile/SOAPnuke.zip). The resulting clean reads were quality controlled by FASTQC again and aligned to the litchi genome (http://litchidb.genomics.cn/) and matched litchi genes (http://litchidb.genomics.cn/, 65,076 sequences) by SOAP2^79^ with no more than a 3-base mismatch. After the number of reads mapped to each gene was counted, the RPKM (reads per kilobase per million reads) method was used for normalization and the lowly expressed genes (<5 RPKM) were filtered in each sample. To identify the DEGs, edgeR^33^ was employed to calculate the log 2-fold change (log2FC), p-value and FDR (false discovery rate) for each gene in every comparison and a strict criterial was used (log2FC > 1 or log2FC < −1, p-value < 0.05 and FDR < 0.05).

### Protein preparation

Proteins were extracted from the litchi fruit tissue (5 g), as previously described^22^. Briefly, the fruit tissue was ground in liquid nitrogen and was homogenized using Buffer A (50 mMTris-HCl pH 8.0, 2 mM EDTA, 100 mMKCl, and 700 mM sucrose). Then, the sample was mixed with an equal volume of Buffer B (Tris-HCl pH 7.5 saturated phenols), homogenized for 3 min on ice and centrifuged at 15,000 rpm for 10 min. Buffer A was used again to extract proteins from the upper organic phase, and ice-cold Buffer C (saturated ammonium acetate in methanol, 4× volume) was then used to precipitate the proteins at −20 °C overnight. The proteins were pelleted by centrifugation, were washed three times with ice-cold Buffer C and were then washed twice in ice-cold acetone. Next, solubilizing buffer (7 M Urea, 2 M Thiourea, 4% CHAPS, 40 mMTris-HCl, pH 8.5, 1 mM PMSF, 2 mM EDTA) was used to suspend the samples, followed by a treatment of sonicate in ice (pulse-on 2 s, pulse-off 3 s, power 180 W). After the proteins were centrifuged at 20,000 rpm for 30 min, they were reduced in 10 mM dithiothreitol (DTT) at 56 °C for 1 h, alkylated by IAM (55 mM) in darkness for 1 h, precipitated in chilled acetone (4 × volume) at −20 °C overnight and centrifuged at 20,000 rpm for 30 min at 4 °C. The pellet was dissolved in 400 µL of 0.5 M TEAB (Applied Biosystems, Milan, Italy) and sonicated in ice for 3 min. After centrifuging at 20,000 rpm for 30 min at 4 °C, the supernatant was collected and the protein concentration was determined by the Bradford method.

### iTRAQ Labelling, SCX fraction and LC-ESI-MS/MS analysis

Proteins (100 µg) of each sample were digested by using Trypsin Gold (Promega) at 37 °C for 16 h (protein: trypsin = 30: 1). Then, peptides were dried by vacuum centrifugation, reconstituted in 0.5 M TEAB and processed with 8-plex iTRAQ labelling reagent according to the manufacturer’s protocol. Samples were labelled by the iTRAQ tags as follows: polLF1d (113), polLF2d (114), polLF4d (115), polLF6d (116), parLF1d (117), parLF2d (118), parLF4d (119) and parLF6d (121). After the peptides were labelled with isobaric tags and were incubated at room temperature for 2 h, they were pooled and subsequently dried by vacuum centrifugation. SCX chromatography was performed with a LC-20AB Pump system (Shimadzu), and tandem mass spectrometry (MS/MS) was performed by a Q EXACTIVE (Thermo Fisher Scientific), as previously described^22^. iTRAQ proteomics analysis was performed twice for all samples.

### Proteome data analysis

Proteome Discoverer 1.2 (Thermo Fisher Scientific) was used to convert the raw data files acquired from the Orbitrap to mascot generic format (MGF) files. The MGF files were used to search against litchi proteins (http://litchidb.genomics.cn/, 65,706 sequences) by Mascot v2.3.02 (Matrix Science). To identify and quantify the proteins in litchi fruits, following parameters were used – quantification: iTRAQ 8-plex; enzyme: trypsin; fixed modification: carbamidomethyl (C), iTRAQ 8-plex (N-term) and iTRAQ 8-plex (K); variable modifications: dioxidation (M), oxidation (M) and iTRAQ 8-plex(Y), mass values: monoisotopic; peptide mass tolerance: ±15 ppm; fragment mass tolerance: ±20 mmu; max missed cleavages: 1; charge states of peptides: +2 and +3. Specifically, an automatic decoy database search was performed in Mascot by choosing the decoy checkbox; in this search a random sequence of the database is generated, and the random sequence and the real database are tested for raw spectra. To reduce the probability of false peptide discovery, peptides with a significant score (≥20) at the 99% confidence interval for a Mascot probability analysis greater than “identity” were counted as identified. In addition, each confident protein identification involves at least one unique peptide. We performed iTRAQ proteomics analysis twice, and the differentially expressed proteins were identified if the ratio was >1.5 in both experiments and the p-value was <0.05, as previously described^22^.

### Functional analysis

To analyse the potential functions of genes and proteins, we first re-annotated the genes of litchi. Briefly, litchi genes and proteins were mapped to multiple public databases such as NCBI non-redundant (NR), Swiss-Prot/UniProt, Gene Ontology (GO) and Kyoto Encyclopedia of Genes and Genomes (KEGG) databases. Using all the genes/proteins as background, we used the numbers of DEGs/proteins to calculate the p-value (<0.05) and q-value (<0.05), which represent the significance of enriched GO terms/KEGG pathways and control the false discovery rate, respectively. The p-values were calculated by Fisher’s exact test and the q-values were calculated by an R package named “qvalue”^80^.

### Weighted gene co-expression network analysis

To identify co-expressed genes and proteins, weighted gene co-expressed network analysis (WGCNA) was applied for both genes and proteins according to the protocol^46^. First, lowly expressed genes (<1 RPKM) were excluded but no restriction was employed for the protein data. Then, we transformed the gene/protein expression into log2(RPKM +1) format, calculated the correlation between samples and performed hierarchical clustering analysis. After the network was constructed and the modules were trained, significant modules and genes were selected for visualization. The modules were filtered using the following criteria: Pearson p > 0.8 and p-value < 0.05. Visualization was performed by using R and Cytoscape.

## Electronic supplementary material

Supplementary Information

Supplementary Dataset

## References

[1] Gillaspy G, Ben-David H, Gruissem W (1993). Fruits: A Developmental Perspective. Plant Cell.

[2] Pascual L, Blanca JM, Canizares J, Nuez F (2009). Transcriptomic analysis of tomato carpel development reveals alterations in ethylene and gibberellin synthesis during pat3/pat4 parthenocarpic fruit set. BMC Plant Biol.

[3] Gorguet B, van Heusden AW, Lindhout P (2005). Parthenocarpic fruit development in tomato. Plant Biol (Stuttg).

[4] de Jong M, Mariani C, Vriezen WH (2009). The role of auxin and gibberellin in tomato fruit set. J Exp Bot.

[5] Watanabe M, Segawa H, Murakami M, Sagawa S, Komori S (2008). Effects of plant growth regulators on fruit set and fruit shape of parthenocarpic apple fruits. Journal of the Japanese Society for Horticultural Science.

[6] Mesejo C, Reig C, Martínez-Fuentes A, Agustí M (2010). Parthenocarpic fruit production in loquat (Eriobotrya japonica Lindl.) by using gibberellic acid. Scientia horticulturae.

[7] Kiyokawa I, Nakagawa S (1972). Parthenocarpic fruit growth and development of the peach as influenced by gibberellin application. Journal of the Japanese Society for Horticultural Science.

[8] Ueguchi-Tanaka M (2005). Gibberellin insensitive dwarf1 encodes a soluble receptor for gibberellin. Nature.

[9] Silverstone AL, Ciampaglio CN, Sun T (1998). The Arabidopsis RGA gene encodes a transcriptional regulator repressing the gibberellin signal transduction pathway. Plant Cell.

[10] Audran-Delalande C (2012). Genome-wide identification, functional analysis and expression profiling of the Aux/IAA gene family in tomato. Plant Cell Physiol.

[11] Zouine M (2014). Characterization of the tomato ARF gene family uncovers a multi-levels post-transcriptional regulation including alternative splicing. PLoS One.

[12] Tiwari SB, Hagen G, Guilfoyle TJ (2004). Aux/IAA proteins contain a potent transcriptional repression domain. Plant Cell.

[13] Guilfoyle TJ, Hagen G (2007). Auxin response factors. Curr Opin Plant Biol.

[14] Chapman EJ, Estelle M (2009). Mechanism of auxin-regulated gene expression in plants. Annual review of genetics.

[15] Wang H (2005). The tomato Aux/IAA transcription factor IAA9 is involved in fruit development and leaf morphogenesis. Plant Cell.

[16] de Jong M, Wolters-Arts M, Garcia-Martinez JL, Mariani C, Vriezen WH (2011). The Solanum lycopersicum Auxin Response Factor 7 (SlARF7) mediates cross-talk between auxin and gibberellin signalling during tomato fruit set and development. J Exp Bot.

[17] Goetz M (2007). Expression of aberrant forms of AUXIN RESPONSE FACTOR8 stimulates parthenocarpy in Arabidopsis and tomato. Plant Physiol.

[18] Tang N, Deng W, Hu G, Hu N, Li Z (2015). Transcriptome profiling reveals the regulatory mechanism underlying pollination dependent and parthenocarpic fruit set mainly mediated by auxin and gibberellin. PLoS One.

[19] de Jong M (2015). Solanum lycopersicum Auxin Response Factor 9 regulates cell division activity during early tomato fruit development. J Exp Bot.

[20] Greening DW, Xu R, Gopal SK, Rai A, Simpson RJ (2017). Proteomic insights into extracellular vesicle biology - defining exosomes and shed microvesicles. Expert review of proteomics.

[21] Chen M (2016). Transcriptome and long noncoding RNA sequencing of three extracellular vesicle subtypes released from the human colon cancer LIM1863 cell line. Sci Rep.

[22] Yang J (2016). iTRAQ-Based Proteomics Identification of Serum Biomarkers of Two Chronic Hepatitis B Subtypes Diagnosed by Traditional Chinese Medicine. Biomed Res Int.

[23] Jiang S, Luo J, Xu F, Zhang X (2016). Transcriptome Analysis Reveals Candidate Genes Involved in Gibberellin-Induced Fruit Setting in Triploid Loquat (Eriobotrya japonica). Front Plant Sci.

[24] Martinez-Esteso MJ, Vilella-Anton MT, Pedreno MA, Valero ML, Bru-Martinez R (2013). iTRAQ-based protein profiling provides insights into the central metabolism changes driving grape berry development and ripening. BMC Plant Biol.

[25] Liu, J. Y., Men, J. L., Chang, M. C., Feng, C. P. & Yuan, L. G. iTRAQ-based quantitative proteome revealed metabolic changes of Flammulina velutipes mycelia in response to cold stress. *J Proteomics*, doi:10.1016/j.jprot.2017.01.009 (2017). 10.1016/j.jprot.2017.01.009 28099886

[26] Guo X, Xu J, Cui X, Chen H, Qi H (2017). iTRAQ-based Protein Profiling and Fruit Quality Changes at Different Development Stages of Oriental Melon. BMC Plant Biol.

[27] Yu T (2016). Proteomic analysis of maize grain development using iTRAQ reveals temporal programs of diverse metabolic processes. BMC Plant Biol.

[28] Li X, Zheng M (2004). Research Progress of Regulatory Mechanism of Flowering and Fruitsetting in Litchi and Its Germplasm Analysis [J]. Chinese Journal of Tropical Agriculture.

[29] Zhang Z, Qiu Y, Xiang X (1990). A Preliminary Report on the Study of Lychee. Fruit Science.

[30] Wang, B. *et al*. Observation on Embryonic and Fruit Development of Lychee Litter. *Journal of Guangdong Agricultural Sciences*, 15–17 (1996).

[31] Wang B, Qiu Y, Xiang X, Yuan P, Zhang Z (1997). Changes of Endogenous Hormones and Induction of Unisexual Results in Litchi Results. acta horticultrae sinica.

[32] Qiu Y, Xiang X (1998). Balance and Fruit Setting Mechanism of Three Kinds of Endogenous Hormones in Litchi. Fruit Science.

[33] Robinson MD, McCarthy DJ, Smyth GK (2010). edgeR: a Bioconductor package for differential expression analysis of digital gene expression data. Bioinformatics.

[34] Yang KZ (2009). A mutation in Thermosensitive Male Sterile 1, encoding a heat shock protein with DnaJ and PDI domains, leads to thermosensitive gametophytic male sterility in Arabidopsis. Plant J.

[35] Wu TY (2013). Interplay between heat shock proteins HSP101 and HSA32 prolongs heat acclimation memory posttranscriptionally in Arabidopsis. Plant Physiol.

[36] Panikulangara TJ, Eggers-Schumacher G, Wunderlich M, Stransky H, Schoffl F (2004). Galactinol synthase1. A novel heat shock factor target gene responsible for heat-induced synthesis of raffinose family oligosaccharides in Arabidopsis. Plant Physiol.

[37] Larance M, Lamond AI (2015). Multidimensional proteomics for cell biology. Nat Rev Mol Cell Biol.

[38] Cheng GW, Breen PJ (1991). Activity of phenylalanine ammonia-lyase (PAL) and concentrations of anthocyanins and phenolics in developing strawberry fruit. Journal of the American Society for Horticultural Science.

[39] Roessner-Tunali U (2003). Metabolic profiling of transgenic tomato plants overexpressing hexokinase reveals that the influence of hexose phosphorylation diminishes during fruit development. Plant Physiol.

[40] Piechulla B, Pichersky E, Cashmore AR, Gruissem W (1986). Expression of nuclear and plastid genes for photosynthesis-specific proteins during tomato fruit development and ripening. Plant Mol Biol.

[41] Gupta, S. *et al*. Identification of Novel Abiotic Stress Proteins in Triticum aestivum Through Functional Annotation of Hypothetical Proteins. *Interdiscip Sci*, doi:10.1007/s12539-016-0178-3 (2016). 10.1007/s12539-016-0178-3 27421996

[42] Minami M, Huh GH, Yang P, Iwabuchi M (1993). Coordinate gene expression of five subclass histones and the putative transcription factors, HBP-1a and HBP-1b, of histone genes in wheat. Plant Mol Biol.

[43] Almeida J, Rocheta M, Galego L (1997). Genetic control of flower shape in Antirrhinum majus. Development.

[44] Beilinson V (2002). Genomic organization of glycinin genes in soybean. Theor Appl Genet.

[45] Van Eldik GJ (1996). Molecular analysis of a pistil-specific gene expressed in the stigma and cortex of Solanum tuberosum. Plant Mol Biol.

[46] Langfelder P, Horvath S (2008). WGCNA: an R package for weighted correlation network analysis. BMC Bioinformatics.

[47] Nambara E, Marion-Poll A (2005). Abscisic acid biosynthesis and catabolism. Annu Rev Plant Biol.

[48] Lundberg E (2010). Defining the transcriptome and proteome in three functionally different human cell lines. Mol Syst Biol.

[49] Rorvig S, Ostergaard O, Heegaard NH, Borregaard N (2013). Proteome profiling of human neutrophil granule subsets, secretory vesicles, and cell membrane: correlation with transcriptome profiling of neutrophil precursors. J Leukoc Biol.

[50] Cabezas-Wallscheid N (2014). Identification of regulatory networks in HSCs and their immediate progeny via integrated proteome, transcriptome, and DNA methylome analysis. Cell Stem Cell.

[51] Kuromori T (2010). ABC transporter AtABCG25 is involved in abscisic acid transport and responses. Proc Natl Acad Sci USA.

[52] Soria-Guerra RE (2011). Gene Expression is Highly Regulated in Early Developing Fruit of Apple. Plant Molecular Biology Reporter.

[53] Pollmann S, Neu D, Weiler EW (2003). Molecular cloning and characterization of an amidase from Arabidopsis thaliana capable of converting indole-3-acetamide into the plant growth hormone, indole-3-acetic acid. Phytochemistry.

[54] Ko T-S, Lee S, Schaefer SC, Korban SS (2003). Characterization of a tissue-specific and developmentally regulated β-1, 3-glucanase gene family in Prunus persica. Plant Physiology and Biochemistry.

[55] Ezquer, I. *et al*. The developmental regulator STK controls the structure and mechanical properties of the Arabidopsis seed coat. *The Plant Cell*, tpc. 00454.02016 (2016). 10.1105/tpc.16.00454 PMC5134981 27624758

[56] Taniguchi Y (2010). Quantifying E. coli proteome and transcriptome with single-molecule sensitivity in single cells. Science.

[57] Griffin TJ (2002). Complementary profiling of gene expression at the transcriptome and proteome levels in Saccharomyces cerevisiae. Mol Cell Proteomics.

[58] Schwabe, W. In *Hortic*. *Abstr*. 661–698.

[59] Koltunow AM, Vivian-Smith A, Tucker MR, Paech N (2002). The central role of the ovule in apomixis and parthenocarpy. Annual Plant Reviews.

[60] de Jong M, Wolters-Arts M, Feron R, Mariani C, Vriezen WH (2009). The Solanum lycopersicum auxin response factor 7 (SlARF7) regulates auxin signaling during tomato fruit set and development. Plant J.

[61] Mesejo C (2016). Gibberellin reactivates and maintains ovary-wall cell division causing fruit set in parthenocarpic Citrus species. Plant Sci.

[62] Davison R (1960). Fruit-setting of apples using gibberellic acid. Nature.

[63] Niu Q (2015). Effects of exogenous application of GA4+7 and N-(2-chloro-4-pyridyl)-N′-phenylurea on induced parthenocarpy and fruit quality in Pyrus pyrifolia ‘Cuiguan’. Plant Growth Regulation.

[64] Yarushnykov VV, Blanke MM (2005). Alleviation of frost damage to pear flowers by application of gibberellin. Plant growth regulation.

[65] Olimpieri I (2007). Tomato fruit set driven by pollination or by the parthenocarpic fruit allele are mediated by transcriptionally regulated gibberellin biosynthesis. Planta.

[66] Garcia-Hurtado N (2012). The characterization of transgenic tomato overexpressing gibberellin 20-oxidase reveals induction of parthenocarpic fruit growth, higher yield, and alteration of the gibberellin biosynthetic pathway. J Exp Bot.

[67] Marti C (2007). Silencing of DELLA induces facultative parthenocarpy in tomato fruits. Plant J.

[68] Carrera E, Ruiz-Rivero O, Peres LE, Atares A, Garcia-Martinez JL (2012). Characterization of the procera tomato mutant shows novel functions of the SlDELLA protein in the control of flower morphology, cell division and expansion, and the auxin-signaling pathway during fruit-set and development. Plant Physiol.

[69] Sun S (2013). GASA14 regulates leaf expansion and abiotic stress resistance by modulating reactive oxygen species accumulation. J Exp Bot.

[70] Tan BC (2003). Molecular characterization of the Arabidopsis 9-cis epoxycarotenoid dioxygenase gene family. Plant J.

[71] Martinelli F (2009). Gene regulation in parthenocarpic tomato fruit. J Exp Bot.

[72] Nagasawa M, Sugiyama A, Mori H, Shiratake K, Yamaki S (2001). Analysis of genes preferentially expressed in early stage of pollinated and parthenocarpic fruit in eggplant. Journal of plant physiology.

[73] Wang W, Vinocur B, Shoseyov O, Altman A (2004). Role of plant heat-shock proteins and molecular chaperones in the abiotic stress response. Trends Plant Sci.

[74] Wei S (2017). Transcriptome Analysis of Taxillusi chinensis (DC.) Danser Seeds in Response to Water Loss. PLoS One.

[75] Vogel JT, Zarka DG, Van Buskirk HA, Fowler SG, Thomashow MF (2005). Roles of the CBF2 and ZAT12 transcription factors in configuring the low temperature transcriptome of Arabidopsis. Plant J.

[76] Davletova S, Schlauch K, Coutu J, Mittler R (2005). The zinc-finger protein Zat12 plays a central role in reactive oxygen and abiotic stress signaling in Arabidopsis. Plant Physiol.

[77] Cini A, Meconcelli S, Cervo R (2013). Ovarian indexes as indicators of reproductive investment and egg-laying activity in social insects: a comparison among methods. Insectes sociaux.

[78] Ji H (2014). Deep sequencing of RNA from three different extracellular vesicle (EV) subtypes released from the human LIM1863 colon cancer cell line uncovers distinct miRNA-enrichment signatures. PLoS One.

[79] Li R (2009). SOAP2: an improved ultrafast tool for short read alignment. Bioinformatics.

[80] Dabney, A., Storey, J. D. & Warnes, G. qvalue: Q-value estimation for false discovery rate control. *R package version* **1** (2010).

