# Supplementary material for: Comprehensive transcriptomics and proteomics analyses of pollinated and parthenocarpic litchi (Litchi chinensis Sonn.) fruits during early development
Source: Sci Rep. 2017 Jul 14;7:5401. doi: 10.1038/s41598-017-05724-z (PMC5511223; doi:10.1038/s41598-017-05724-z)
Supplement: Supplementary file 1 — Supplementary Information [file 41598_2017_5724_MOESM1_ESM.pdf]

Supplementary Information

Comprehensive transcriptomics and proteomics analyses of pollinated and parthenocarpic litchi (*Litchi chinensis* Sonn.) fruits during early development

Wei Liu [1,2,3,†], Maoshan Chen[4,†], Lijun Bai [5], Zhenhua Zhuang[5], Chao Fan[1,2,3], Nonghui Jiang [1,2,3], Junsheng Zhao [6], Shuaipeng Ma [7], Xu Xiang [1,2,3,*]

[1]Institute of Fruit Tree Research, Guangdong Academy of Agricultural Sciences, Guangzhou 510640, China.

[2]Key Laboratory of South Subtropical Fruit Biology and Genetic Resource Utilization, Ministry of Agriculture, Guangzhou 510640, China.

[3]Guangdong Provincial Key Laboratory of Tropical and Subtropical Fruit Tree Research, Guangzhou 510640, China.

[4]Department of Biochemistry and Genetics, La Trobe Institute for Molecular Science (LIMS), La Trobe University, Melbourne, Victoria 3086, Australia

[5]Chengdu Life Baseline Technology, Chengdu 610041, China

[6]Institute of Fruit Science in Maoming, Maoming 525000, China

[7]Guangzhou Experiment Station of Chinese Academy of Tropical Agricultural Sciences, Guangzhou 510140, China

[†]These authors contribute equally to this work

***Correspondence to**:

Xu Xiang PhD

Institute of Fruit Tree Research

Guangdong Academy of Agricultural Sciences

80 Da Feng Second Street, Tianhe District

Guangzhou 510640, China

Tel: +86 20 3876 5789

Email: xiangxu@vip.163.com

Keywords: parthenocarpy, transcriptome, litchi, fruit development, pollination

Fig. S1. Distribution of genes identified in polLF and parLF.

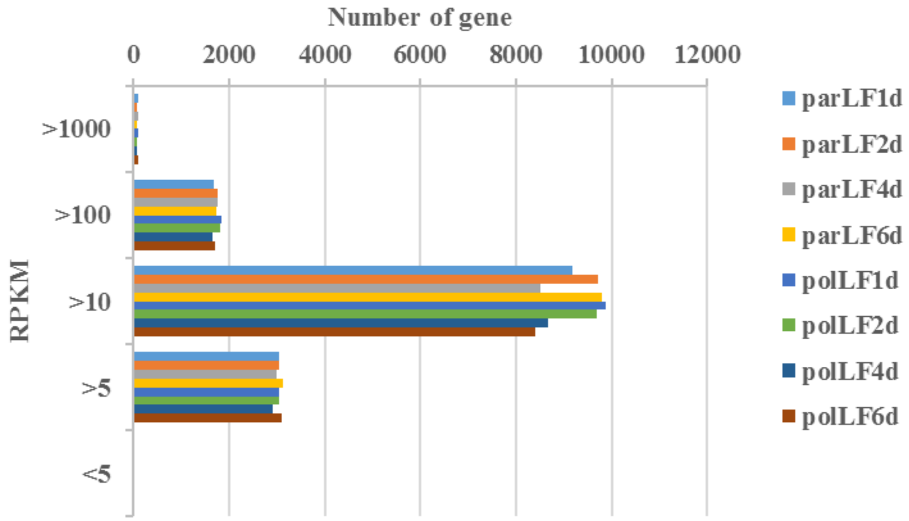

Fig. S2. Venn diagrams of DEPs in polLFs and parLFs at different stages.

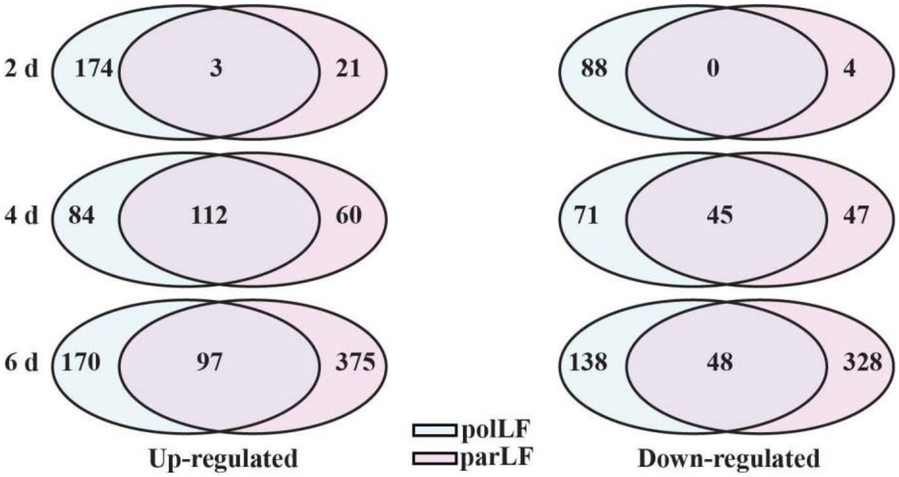

Fig. S3. Scatter plot of consistent DEGs/DEPs in parLF6d compared to polLF6d identified by both transcriptome sequencing and iTRAQ.

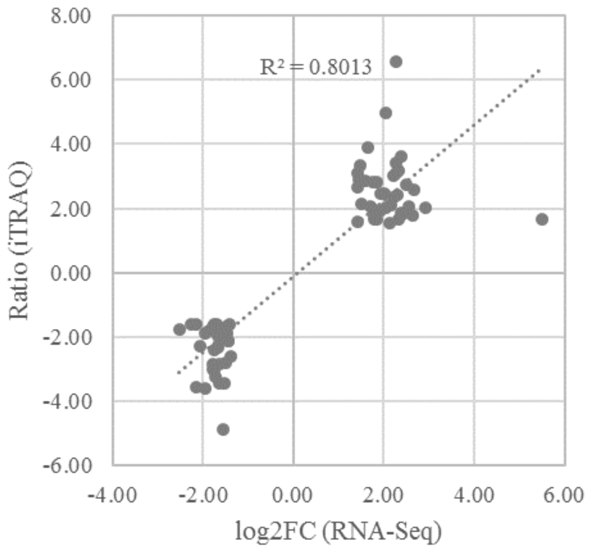

Table S1. KEGG pathway enrichment of DEGs in polLFs and parLFs

Group	KEGG pathway	KO_id	polLFs[a]	q-value[c]	parLFs[b]	q-value[c]
Global and overview maps	Metabolic pathways	ko01100	991	7.57E-14	783	7.44E-14
Global and overview maps	Biosynthesis of secondary metabolites	ko01110	668	7.57E-14	565	7.44E-14
Global and overview maps	Microbial metabolism in diverse environments	ko01120	192	8.92E-08	155	2.08E-07
Global and overview maps	Biosynthesis of antibiotics	ko01130	194	1.53E-11	154	7.54E-10
Translation	Ribosome	ko03010	216	7.57E-14	150	7.44E-14
Signal transduction	Plant hormone signal transduction	ko04075	165	2.21E-10	132	4.13E-09
Global and overview maps	Carbon metabolism	ko01200	124	4.72E-08	99	6.12E-07
Biosynthesis of other secondary metabolites	Phenylpropanoid biosynthesis	ko00940	113	0.000654	92	0.000658
Folding, sorting and degradation	Protein processing in endoplasmic reticulum	ko04141	88	0.184487	90	1.93E-06
Biosynthesis of other secondary metabolites	Flavonoid biosynthesis	ko00941	72	1.32E-07	67	1.67E-10
Carbohydrate metabolism	Pentose and glucuronate interconversions	ko00040	66	0.00989	64	5.75E-06
Carbohydrate metabolism	Glycolysis / Gluconeogenesis	ko00010	76	1.33E-05	57	0.001597
Biosynthesis of other secondary metabolites	Stilbenoid, diarylheptanoid and gingerol biosynthesis	ko00945	53	0.033826	51	0.000105
Transport and catabolism	Phagosome	ko04145	56	1.27E-06	47	1.89E-06
Cell motility	Regulation of actin cytoskeleton	ko04810	53	2.32E-07	45	2.64E-07
Xenobiotics biodegradation and metabolism	Metabolism of xenobiotics by cytochrome P450	ko00980	42	0.002648	44	2.65E-07
Environmental adaptation	Circadian rhythm - plant	ko04712	53	0.000148	43	0.00052
Carbohydrate metabolism	Pyruvate metabolism	ko00620	56	7.52E-08	41	9.3E-05
Lipid metabolism	Glycerolipid metabolism	ko00561	44	0.44892	41	0.013469
Endocrine system	Glucagon signaling pathway	ko04922	59	1.39E-06	40	0.010184
Endocrine system	Estrogen signaling pathway	ko04915	43	0.52804	40	0.018137
Carbohydrate metabolism	Galactose metabolism	ko00052	51	0.003143	39	0.042081
Global and overview maps	Fatty acid metabolism	ko01212	49	2.77E-06	39	4.59E-05
Transport and catabolism	Peroxisome	ko04146	44	0.004035	37	0.003133
Xenobiotics biodegradation and metabolism	Drug metabolism - cytochrome P450	ko00982	34	0.581016	37	0.000257
Lipid metabolism	alpha-Linolenic acid metabolism	ko00592	46	6.7E-08	36	5.22E-06
Metabolism of other amino acids	Glutathione metabolism	ko00480	32	0.401104	32	0.003427
Metabolism of terpenoids and polyketides	Terpenoid backbone biosynthesis	ko00900	32	1	32	0.016755
Energy metabolism	Carbon fixation in photosynthetic organisms	ko00710	39	0.007644	31	0.030913
Carbohydrate metabolism	Ascorbate and aldarate metabolism	ko00053	36	0.000165	29	0.000984
Amino acid metabolism	Valine, leucine and isoleucine degradation	ko00280	35	2.45E-07	28	6.96E-06
Lipid metabolism	Fatty acid degradation	ko00071	38	8.11E-11	28	4.99E-07
Signal transduction	HIF-1 signaling pathway	ko04066	35	0.001218	26	0.041912
Signal transduction	Rap1 signaling pathway	ko04015	33	0.000697	25	0.017177
Digestive system	Mineral absorption	ko04978	31	4.87E-09	23	6.35E-06
Carbohydrate metabolism	Propanoate metabolism	ko00640	27	7.6E-06	21	0.000292
Lipid metabolism	Fatty acid biosynthesis	ko00061	26	0.003509	21	0.013442
Lipid metabolism	Biosynthesis of unsaturated fatty acids	ko01040	21	0.001731	19	0.000505
Endocrine system	PPAR signaling pathway	ko03320	22	0.000463	17	0.008075
Metabolism of terpenoids and polyketides	Monoterpenoid biosynthesis	ko00902	15	1	16	0.036335
Lipid metabolism	Linoleic acid metabolism	ko00591	18	0.016223	15	0.030805
Signaling molecules and interaction	Cell adhesion molecules (CAMs)	ko04514	11	0.005941	10	0.003674
Biosynthesis of other secondary metabolites	Aflatoxin biosynthesis	ko00254	7	0.021132	6	0.045292
Amino acid metabolism	Cysteine and methionine metabolism	ko00270	46	0.000847	30	0.730756
Cellular community	Focal adhesion	ko04510	24	0.004059	17	0.216455
Endocrine system	Insulin signaling pathway	ko04910	44	0.002621	31	0.269961
Energy metabolism	Photosynthesis - antenna proteins	ko00196	11	0.00097	6	1
Glycan biosynthesis and metabolism	Other glycan degradation	ko00511	48	0.011565	37	0.090922
Signal transduction	cAMP signaling pathway	ko04024	44	1.28E-05	27	0.257691
Signaling molecules and interaction	ECM-receptor interaction	ko04512	14	0.001302	10	0.07196

[a]Number of DEGs in polLFs.

[b]Number of DEGs in parLFs.

[c]adjusted p-values.

Table S2. Gene Ontology analysis of differentially expressed genes in polLFs

Category	GO term	Gene number	p-value	q-value[a]
Biological process	GO:0035335~peptidyl-tyrosine dephosphorylation	8	2.2E-16	3.16E-13
	GO:0016485~protein processing	6	2.3E-09	3.31E-06
	GO:0006098~pentose-phosphate shunt	19	1.46E-08	2.1E-05
	GO:0080051~cutin transport	2	3.631E-06	0.005218
	GO:0044805~late nucleophagy	1	3.763E-06	0.005407
	GO:0080129~proteasome core complex assembly	13	3.782E-06	0.005435
	GO:0007346~regulation of mitotic cell cycle	5	5.594E-06	0.008039
	GO:0033356~UDP-L-arabinose metabolic process	5	6.272E-06	0.009013
	GO:0009639~response to red or far red light	1	6.281E-06	0.009026
	GO:0006662~glycerol ether metabolic process	10	1.016E-05	0.0146
	GO:0019243~methylglyoxal catabolic process to D-lactate via S-lactoyl-glutathione	8	1.238E-05	0.01779
	GO:0007094~mitotic spindle assembly checkpoint	1	1.922E-05	0.027619
	GO:0019432~triglyceride biosynthetic process	1	2.265E-05	0.032548
Molecular function	GO:0043621~protein self-association	1	2.2E-16	1.95E-13
	GO:0003678~DNA helicase activity	4	3.788E-07	0.000335
	GO:0008113~peptide-methionine (S)-S-oxide reductase activity	1	6.908E-07	0.000611
	GO:0003988~acetyl-CoA C-acyltransferase activity	1	6.032E-06	0.005338
	GO:0004471~malate dehydrogenase (decarboxylating) (NAD+) activity	2	1.794E-05	0.015877
	GO:0008792~arginine decarboxylase activity	2	5.601E-05	0.049569
Cellular component	GO:0022625~cytosolic large ribosomal subunit	56	2.2E-16	5.76E-14
	GO:0022627~cytosolic small ribosomal subunit	32	3.273E-13	8.58E-11
	GO:0009505~plant-type cell wall	61	1.024E-12	2.68E-10
	GO:0046658~anchored component of plasma membrane	35	2.995E-10	7.85E-08
	GO:0000786~nucleosome	21	6.044E-08	1.58E-05
	GO:0005840~ribosome	53	2.086E-07	5.47E-05
	GO:0048046~apoplast	60	3.635E-07	9.52E-05
	GO:0009506~plasmodesma	110	3.915E-05	0.010257
	GO:0005618~cell wall	80	9.073E-05	0.023771
	GO:0015935~small ribosomal subunit	10	0.0001054	0.027615

[a]adjusted p-values.

Table S3. Gene Ontology analysis of differentially expressed genes in parLFs

Category	GO term	Gene number	p-value	q-value[a]
Biological process	GO:0035428~hexose transmembrane transport	1	2.2E-16	2.71E-13
	GO:0051457~maintenance of protein location in nucleus	1	1.26E-14	1.54E-11
	GO:0015780~nucleotide-sugar transport	1	3.93E-09	4.84E-06
	GO:0051555~flavonol biosynthetic process	5	1.06E-06	0.001305
	GO:0052033~pathogen-associated molecular pattern dependent induction by symbiont of host innate immune response	2	1.21E-06	0.001487
	GO:0050896~response to stimulus	21	2.56E-06	0.003152
	GO:0071310~cellular response to organic substance	6	3.36E-06	0.004138
	GO:0090480~purine nucleotide-sugar transmembrane transport	1	5.6E-06	0.006886
	GO:0035999~tetrahydrofolate interconversion	1	1.11E-05	0.013592
	GO:0016125~sterol metabolic process	4	2.35E-05	0.02893
	GO:0010304~PSII associated light-harvesting complex II catabolic process	2	3.31E-05	0.040725
	GO:0051603~proteolysis involved in cellular protein catabolic process	8	3.51E-05	0.043136
Molecular function	GO:0004743~pyruvate kinase activity	4	2.2E-16	1.65E-13
	GO:0003746~translation elongation factor activity	7	1.6E-07	0.000119
	GO:0001671~ATPase activator activity	2	3.63E-07	0.000272
	GO:0004471~malate dehydrogenase (decarboxylating) (NAD+) activity	2	4.68E-07	0.00035
	GO:0032050~clathrin heavy chain binding	1	5.23E-06	0.003914
	GO:0001653~peptide receptor activity	1	2E-05	0.014923
	GO:0047714~galactolipase activity	1	2.14E-05	0.01603
	GO:0003922~GMP synthase (glutamine-hydrolyzing) activity	2	2.51E-05	0.018805
Cellular component	GO:0022625~cytosolic large ribosomal subunit	41	1.65E-13	3.47E-11
	GO:0046658~anchored component of plasma membrane	32	1.84E-11	3.86E-09
	GO:0022627~cytosolic small ribosomal subunit	25	1.56E-10	3.28E-08
	GO:0009505~plant-type cell wall	47	4.13E-10	8.67E-08
	GO:0000786~nucleosome	20	2.96E-09	6.22E-07
	GO:0048046~apoplast	51	1.01E-07	2.12E-05
	GO:0005618~cell wall	72	6.62E-07	0.000139
	GO:0005576~extracellular region	76	1.16E-06	0.000243
	GO:0015935~small ribosomal subunit	9	6.98E-05	0.014666
	GO:0005840~ribosome	36	0.000219	0.046053

[a]adjusted p-values.

Table S4. KEGG pathway enrichment of common DEPs in polLFs and parLFs

Group	Pathway	ID	Protein_number	q-value[a]
Global and overview maps	Metabolic pathways	ko01100	104	3.43E-14
Global and overview maps	Biosynthesis of secondary metabolites	ko01110	64	2.12E-10
Biosynthesis of other secondary metabolites	Phenylpropanoid biosynthesis	ko00940	20	5.76E-06
Metabolism of cofactors and vitamins	Ubiquinone and other terpenoid-quinone biosynthesis	ko00130	7	1.00E-03
Energy metabolism	Photosynthesis	ko00195	6	1.66E-03
Carbohydrate metabolism	Amino sugar and nucleotide sugar metabolism	ko00520	12	3.13E-03
Amino acid metabolism	Phenylalanine metabolism	ko00360	8	3.70E-03
Biosynthesis of other secondary metabolites	Aflatoxin biosynthesis	ko00254	3	7.25E-03
Carbohydrate metabolism	Pyruvate metabolism	ko00620	8	1.47E-02
Glycan biosynthesis and metabolism	Other glycan degradation	ko00511	8	4.12E-02
Lipid metabolism	Sphingolipid metabolism	ko00600	6	4.23E-02

[a]adjusted p-values.

Table S5. KEGG pathway enrichment of DEPs identified exclusively in parLFs

Group	Pathway	ID	Protein_number	q-value[a]
Global and overview maps	Metabolic pathways	ko01100	130	3.29E-11
Transport and catabolism	Lysosome	ko04142	16	1.46E-05
Glycan biosynthesis and metabolism	Other glycan degradation	ko00511	15	1.13E-04
Translation	Ribosome	ko03010	21	1.39E-03
Glycan biosynthesis and metabolism	Glycosaminoglycan degradation	ko00531	8	2.46E-03
Global and overview maps	Carbon metabolism	ko01200	22	4.88E-03
Transport and catabolism	Phagosome	ko04145	12	6.64E-03
Glycan biosynthesis and metabolism	Glycosphingolipid biosynthesis - ganglio series	ko00604	7	1.02E-02
Endocrine system	Thyroid hormone synthesis	ko04918	5	2.13E-02
Energy metabolism	Carbon fixation in photosynthetic organisms	ko00710	10	2.15E-02
Folding, sorting and degradation	Protein processing in endoplasmic reticulum	ko04141	19	2.96E-02
Biosynthesis of other secondary metabolites	Phenylpropanoid biosynthesis	ko00940	21	3.02E-02
Global and overview maps	Biosynthesis of antibiotics	ko01130	28	3.12E-02
Signaling molecules and interaction	Cell adhesion molecules (CAMs)	ko04514	4	4.02E-02

[a]adjusted p-values.

Table S6. KEGG pathway enrichment of DEPs identified parLFs compared to polLFs

Stage	Group	Pathway	ID	Protein_number	q-value[a]
2d	Global and overview maps	Metabolic pathways	ko01100	163	5.72E-14
	Global and overview maps	Biosynthesis of secondary metabolites	ko01110	102	1.04E-05
	Transcription	Spliceosome	ko03040	24	3.35E-04
	Biosynthesis of other secondary metabolites	Phenylpropanoid biosynthesis	ko00940	27	2.36E-03
	Energy metabolism	Photosynthesis	ko00195	8	4.96E-03
	Carbohydrate metabolism	Amino sugar and nucleotide sugar metabolism	ko00520	18	1.75E-02
	Global and overview maps	Carbon metabolism	ko01200	24	2.05E-02
	Global and overview maps	Biosynthesis of antibiotics	ko01130	33	2.80E-02
	Transport and catabolism	Peroxisome	ko04146	12	2.83E-02
4d	Translation	RNA transport	ko03013	8	0.002219
6d	Global and overview maps	Metabolic pathways	ko01100	256	6.60E-14
	Global and overview maps	Biosynthesis of secondary metabolites	ko01110	146	9.54E-11
	Global and overview maps	Carbon metabolism	ko01200	44	2.24E-09
	Global and overview maps	Biosynthesis of antibiotics	ko01130	57	1.62E-08
	Global and overview maps	Microbial metabolism in diverse environments	ko01120	57	3.81E-07
	Lipid metabolism	Fatty acid degradation	ko00071	14	2.45E-06
	Carbohydrate metabolism	Pyruvate metabolism	ko00620	18	1.58E-04
	Energy metabolism	Carbon fixation in photosynthetic organisms	ko00710	16	4.44E-04
	Carbohydrate metabolism	Amino sugar and nucleotide sugar metabolism	ko00520	25	4.70E-04
	Energy metabolism	Carbon fixation pathways in prokaryotes	ko00720	10	4.86E-04
	Metabolism of cofactors and vitamins	Ubiquinone and other terpenoid-quinone biosynthesis	ko00130	11	3.21E-03
	Transport and catabolism	Peroxisome	ko04146	15	1.07E-02
	Energy metabolism	Photosynthesis - antenna proteins	ko00196	5	1.38E-02
	Glycan biosynthesis and metabolism	Other glycan degradation	ko00511	16	1.87E-02
	Amino acid metabolism	Alanine, aspartate and glutamate metabolism	ko00250	12	1.87E-02
	Energy metabolism	Methane metabolism	ko00680	14	2.11E-02
	Biosynthesis of other secondary metabolites	Phenylpropanoid biosynthesis	ko00940	30	3.20E-02
	Carbohydrate metabolism	Glyoxylate and dicarboxylate metabolism	ko00630	15	3.83E-02
	Energy metabolism	Photosynthesis	ko00195	8	4.51E-02
	Carbohydrate metabolism	Ascorbate and aldarate metabolism	ko00053	11	4.51E-02

[a]adjusted p-values.

Table S7. KEGG pathway enrichment of co-expressed genes in parLFs and polLFs

Module	Group	Pathway	ID	Gene_number	q-value[a]
Brown	Global and overview maps	Metabolic pathways	ko01100	331	9.48E-14
	Environmental adaptation	Circadian rhythm - plant	ko04712	31	1.61E-06
	Folding, sorting and degradation	Proteasome	ko03050	24	3.85E-06
	Global and overview maps	Biosynthesis of antibiotics	ko01130	75	6.72E-05
	Global and overview maps	Microbial metabolism in diverse environments	ko01120	77	4.37E-04
	Energy metabolism	Oxidative phosphorylation	ko00190	22	1.48E-03
	Global and overview maps	Biosynthesis of secondary metabolites	ko01110	201	1.86E-03
	Excretory system	Vasopressin-regulated water reabsorption	ko04962	11	5.18E-03
	Xenobiotics biodegradation and metabolism	Chloroalkane and chloroalkene degradation	ko00625	10	7.78E-03
	Global and overview maps	Biosynthesis of amino acids	ko01230	47	1.06E-02
	Amino acid metabolism	Phenylalanine metabolism	ko00360	19	1.33E-02
	Amino acid metabolism	Tyrosine metabolism	ko00350	20	2.52E-02
	Folding, sorting and degradation	Protein processing in endoplasmic reticulum	ko04141	41	4.48E-02
Pink	Global and overview maps	Biosynthesis of secondary metabolites	ko01110	103	5.81E-14
	Global and overview maps	Metabolic pathways	ko01100	133	5.81E-14
	Metabolism of terpenoids and polyketides	Sesquiterpenoid and triterpenoid biosynthesis	ko00909	21	1.36E-10
	Metabolism of cofactors and vitamins	Ubiquinone and other terpenoid-quinone biosynthesis	ko00130	10	6.44E-05
	Global and overview maps	Biosynthesis of antibiotics	ko01130	26	3.39E-02
	Biosynthesis of other secondary metabolites	Anthocyanin biosynthesis	ko00942	6	4.72E-02

[a]adjusted p-values.

Table S8. KEGG pathway enrichment of co-expressed proteins in parLFs and polLFs

Module	Group	Pathway	ID	Protein_number	q-value[a]
Blue	Global and overview maps	Metabolic pathways	ko01100	269	6.42E-14
	Global and overview maps	Biosynthesis of antibiotics	ko01130	69	5.86E-10
	Global and overview maps	Biosynthesis of secondary metabolites	ko01110	165	1.79E-08
	Carbohydrate metabolism	Amino sugar and nucleotide sugar metabolism	ko00520	31	2.91E-05
	Metabolism of other amino acids	Glutathione metabolism	ko00480	18	1.56E-04
	Global and overview maps	Biosynthesis of amino acids	ko01230	38	1.19E-03
	Metabolism of cofactors and vitamins	Pantothenate and CoA biosynthesis	ko00770	12	2.41E-03
	Global and overview maps	Microbial metabolism in diverse environments	ko01120	55	2.81E-03
	Carbohydrate metabolism	Galactose metabolism	ko00052	20	3.48E-03
	Global and overview maps	Carbon metabolism	ko01200	36	4.90E-03
	Glycan biosynthesis and metabolism	Glycosphingolipid biosynthesis - globo series	ko00603	7	6.95E-03
	Carbohydrate metabolism	Pyruvate metabolism	ko00620	17	1.17E-02
	Biosynthesis of other secondary metabolites	Phenylpropanoid biosynthesis	ko00940	36	1.46E-02
	Glycan biosynthesis and metabolism	Glycosaminoglycan degradation	ko00531	10	2.11E-02
	Amino acid metabolism	Valine, leucine and isoleucine degradation	ko00280	11	2.30E-02
	Translation	RNA transport	ko03013	32	3.80E-02
Brown	Global and overview maps	Biosynthesis of antibiotics	ko01130	73	6.42E-14
	Global and overview maps	Metabolic pathways	ko01100	217	6.42E-14
	Global and overview maps	Biosynthesis of amino acids	ko01230	51	3.96E-12
	Global and overview maps	Microbial metabolism in diverse environments	ko01120	63	1.43E-08
	Global and overview maps	Carbon metabolism	ko01200	43	7.13E-08
	Transcription	Spliceosome	ko03040	37	8.58E-08
	Translation	RNA transport	ko03013	40	3.85E-07
	Global and overview maps	Biosynthesis of secondary metabolites	ko01110	139	2.37E-06
	Folding, sorting and degradation	Protein processing in endoplasmic reticulum	ko04141	33	3.25E-04
	Amino acid metabolism	Cysteine and methionine metabolism	ko00270	18	3.83E-04
	Energy metabolism	Carbon fixation pathways in prokaryotes	ko00720	10	8.77E-04
	Carbohydrate metabolism	Pyruvate metabolism	ko00620	17	1.73E-03
	Endocrine and metabolic diseases	Type II diabetes mellitus	ko04930	8	1.96E-03
	Amino acid metabolism	Phenylalanine, tyrosine and tryptophan biosynthesis	ko00400	11	1.04E-02
	Amino acid metabolism	Histidine metabolism	ko00340	6	1.34E-02
	Translation	Ribosome biogenesis in eukaryotes	ko03008	17	3.65E-02
	Energy metabolism	Sulfur metabolism	ko00920	8	3.71E-02
Green	Global and overview maps	Metabolic pathways	ko01100	91	2.09E-08
	Global and overview maps	Biosynthesis of antibiotics	ko01130	31	3.41E-07
	Translation	Ribosome	ko03010	19	5.97E-05
	Global and overview maps	Biosynthesis of secondary metabolites	ko01110	59	9.08E-04
	Global and overview maps	Microbial metabolism in diverse environments	ko01120	24	9.79E-03
	Carbohydrate metabolism	Butanoate metabolism	ko00650	5	3.13E-02
	Global and overview maps	Biosynthesis of amino acids	ko01230	16	3.67E-02
Yellow	Global and overview maps	Metabolic pathways	ko01100	134	8.77E-10
	Global and overview maps	Carbon metabolism	ko01200	33	1.70E-08
	Global and overview maps	Biosynthesis of antibiotics	ko01130	38	9.32E-06
	Global and overview maps	Microbial metabolism in diverse environments	ko01120	37	2.20E-04
	Energy metabolism	Oxidative phosphorylation	ko00190	13	5.48E-04
	Global and overview maps	Biosynthesis of secondary metabolites	ko01110	83	2.66E-03
	Folding, sorting and degradation	Proteasome	ko03050	11	2.93E-03
	Endocrine and metabolic diseases	Type I diabetes mellitus	ko04940	5	5.94E-03
	Endocrine and metabolic diseases	Non-alcoholic fatty liver disease (NAFLD)	ko04932	9	1.51E-02
	Global and overview maps	Biosynthesis of amino acids	ko01230	22	2.34E-02
	Signal transduction	FoxO signaling pathway	ko04068	9	3.83E-02

[a]adjusted p-values.

Table S9. Consistent DEGs/DEPs in parLFs compared to polLFs

ID	RNA-Seq[a]	iTRAQ[b]	pro_id	description
Litchi_GLEAN_10061682	5.51	1.67	XCP1_ARATH	Xylem cysteine proteinase 1
Litchi_GLEAN_10049566	2.93	2.01	PER72_ARATH	Peroxidase 72
Litchi_GLEAN_10065071	2.66	2.60	FH2_ARATH	Formin-like protein 2
Litchi_GLEAN_10020874	2.65	1.79	ASO_TOBAC	L-ascorbate oxidase
Litchi_GLEAN_10003635	2.56	2.07	4CL2_SOYBN	4-coumarate--CoA ligase 2
Litchi_GLEAN_10058276	2.49	2.74	RLP12_ARATH	Receptor-like protein 12
Litchi_GLEAN_10031213	2.41	1.82	C3H53_ORYSJ	Zinc finger CCCH domain-containing protein 53
Litchi_GLEAN_10008357	2.39	1.86	E1313_ARATH	Glucan endo-1,3-beta-glucosidase 13
Litchi_GLEAN_10038360	2.38	3.62	DUT_ARATH	Deoxyuridine 5'-triphosphate nucleotidohydrolase
Litchi_GLEAN_10036690	2.34	3.19	SUBL_ARATH	Subtilisin-like protease
Litchi_GLEAN_10053304	2.32	1.65	LYM1_ARATH	LysM domain-containing GPI-anchored protein 1
Litchi_GLEAN_10049413	2.30	2.43	EXOL3_ARATH	Protein EXORDIUM-like 3
Litchi_GLEAN_10025692	2.27	6.56	LAC14_ARATH	Laccase-14
Litchi_GLEAN_10039316	2.26	3.42	GPDA_ANAD2	Glycerol-3-phosphate dehydrogenase [NAD(P)+]
Litchi_GLEAN_10006890	2.26	3.12	SUBL_ARATH	Subtilisin-like protease
Litchi_GLEAN_10027616	2.22	3.02	FLA2_ARATH	Fasciclin-like arabinogalactan protein 2
Litchi_GLEAN_10041901	2.21	2.35	Y5258_ARATH	Uncharacterized protein At5g22580
Litchi_GLEAN_10009972	2.17	2.08	SKU5_ARATH	Monocopper oxidase-like protein SKU5
Litchi_GLEAN_10028285	2.12	1.54	ASOL_BRANA	L-ascorbate oxidase homolog
Litchi_GLEAN_10014033	2.05	2.01	CFI3_ARATH	Probable chalcone--flavonone isomerase 3
Litchi_GLEAN_10032621	2.04	4.99	PALY_POPTR	Phenylalanine ammonia-lyase
Litchi_GLEAN_10039644	2.01	2.46	CHS1_CAMSI	Chalcone synthase 1
Litchi_GLEAN_10055014	1.93	1.92	FL3H_PETHY	Naringenin,2-oxoglutarate 3-dioxygenase
Litchi_GLEAN_10063889	1.93	2.45	INVB_DAUCA	Beta-fructofuranosidase, soluble isoenzyme I
Litchi_GLEAN_10015949	1.84	2.81	GUN10_ARATH	Endoglucanase 10
Litchi_GLEAN_10019144	1.84	1.67	SUBL_ARATH	Subtilisin-like protease
Litchi_GLEAN_10007383	1.79	1.73	LAR_DESUN	Leucoanthocyanidin reductase
Litchi_GLEAN_10015524	1.78	1.67	PATL6_ARATH	Patellin-6
Litchi_GLEAN_10024056	1.77	2.83	PRP1_ARATH	Proline-rich protein 1
Litchi_GLEAN_10065046	1.76	1.96	XTH2_SOYBN	Xyloglucan endotransglucosylase/hydrolase 2
Litchi_GLEAN_10003238	1.70	2.06	PDCB3_ARATH	PLASMODESMATA CALLOSE-BINDING PROTEIN 3
Litchi_GLEAN_10014880	1.65	3.88	ALDOL_ORYSJ	Putative aldehyde oxidase-like protein
Litchi_GLEAN_10021245	1.59	2.85	Y4744_ARATH	Uncharacterized protein At4g06744
Litchi_GLEAN_10007655	1.51	2.13	LRX2_ARATH	Leucine-rich repeat extensin-like protein 2
Litchi_GLEAN_10025986	1.47	3.33	XTH9_ARATH	Xyloglucan endotransglucosylase/hydrolase protein 9
Litchi_GLEAN_10042347	1.45	2.89	ENL1_ARATH	Early nodulin-like protein 1
Litchi_GLEAN_10039876	1.43	2.66	ORYA_ORYSJ	Oryzain alpha chain
Litchi_GLEAN_10064182	1.42	1.58	CFI_CITSI	Chalcone--flavonone isomerase
Litchi_GLEAN_10023978	1.41	3.12	INO1_SESIN	Inositol-3-phosphate synthase
Litchi_GLEAN_10053488	-1.39	-2.61	CHI4_ORYSJ	Chitinase 4
Litchi_GLEAN_10007037	-1.41	-1.62	7SB1_SOYBN	Basic 7S globulin
Litchi_GLEAN_10038226	-1.43	-2.11	E13B_PRUPE	Glucan endo-1,3-beta-glucosidase, basic isoform
Litchi_GLEAN_10054512	-1.43	-2.13	DHEA_NICPL	Glutamate dehydrogenase A
Litchi_GLEAN_10052962	-1.47	-1.87	WTR37_ARATH	WAT1-related protein At4g30420
Litchi_GLEAN_10028148	-1.49	-1.67	BGAL_SOLLC	Beta-galactosidase
Litchi_GLEAN_10049423	-1.50	-2.81	TLP_ACTDE	Thaumatin-like protein
Litchi_GLEAN_10010659	-1.54	-3.44	BXL1_ARATH	Beta-D-xylosidase 1
Litchi_GLEAN_10043707	-1.54	-4.87	GLYG3_SOYBN	Glycinin G3
Litchi_GLEAN_10048452	-1.59	-2.10	LAC7_ARATH	Laccase-7
Litchi_GLEAN_10057272	-1.62	-2.84	MBCD_SOLTU	2-methylacyl-CoA dehydrogenase, mitochondrial
Litchi_GLEAN_10016777	-1.64	-3.42	SUT31_ARATH	Sulfate transporter 3.1
Litchi_GLEAN_10053051	-1.64	-1.84	2NPD_BACSU	Probable nitronate monooxygenase
Litchi_GLEAN_10043236	-1.66	-1.96	BSP_BOSSE	Basic secretory protease
Litchi_GLEAN_10007077	-1.66	-2.32	KING1_ARATH	SNF1-related protein kinase regulatory subunit gamma-1
Litchi_GLEAN_10059810	-1.68	-1.60	LIRP1_ORYSJ	Light-regulated protein
Litchi_GLEAN_10049847	-1.69	-1.63	PGML1_ARATH	Phosphoglycerate mutase-like protein 1

Litchi_GLEAN_10030329	-1.73	-3.22	PRPX_HORVU	Pathogen-related protein
Litchi_GLEAN_10007251	-1.75	-3.02	ACCH1_ARATH	1-aminocyclopropane-1-carboxylate oxidase homolog 1
Litchi_GLEAN_10006178	-1.76	-1.59	DBR_TOBAC	2-alkenal reductase (NADP(+)-dependent)
Litchi_GLEAN_10018848	-1.76	-2.39	E13B_HEVBR	Glucan endo-1,3-beta-glucosidase, basic vacuolar isoform
Litchi_GLEAN_10060520	-1.78	-3.00	AL2B4_ARATH	Aldehyde dehydrogenase family 2 member B4, mitochondrial
Litchi_GLEAN_10040929	-1.78	-1.68	PERX_NICSY	Lignin-forming anionic peroxidase
Litchi_GLEAN_10012603	-1.78	-2.82	RETOL_ARATH	Reticuline oxidase-like protein
Litchi_GLEAN_10057113	-1.86	-1.80	TLP1_PRUPE	Thaumatin-like protein 1
Litchi_GLEAN_10001487	-1.93	-1.89	GATY_SALTY	D-tagatose-1,6-bisphosphate aldolase subunit GatY
Litchi_GLEAN_10012104	-1.95	-3.58	ITH5_CUCMA	Inhibitor of trypsin and hageman factor
Litchi_GLEAN_10011351	-2.07	-2.29	MIRA_SYNDU	Miraculin
Litchi_GLEAN_10037528	-2.14	-1.60	IF2P_SCHPO	Eukaryotic translation initiation factor 5B
Litchi_GLEAN_10062296	-2.14	-3.55	TRNH1_ARATH	Tropinone reductase homolog At1g07440
Litchi_GLEAN_10063755	-2.27	-1.59	LEC_PARPC	Mannose/glucose-specific lectin
Litchi_GLEAN_10012605	-2.52	-1.75	PSBA_PANGI	Photosystem II protein D1
